# ESRP1-driven alternative splicing of CLSTN1 inhibits the metastasis of gastric cancer

**DOI:** 10.1038/s41420-023-01757-8

**Published:** 2023-12-19

**Authors:** Chengguo Li, Yuping Yin, Ruikang Tao, Yao Lin, Tao Wang, Qian Shen, Runze Li, Kaixiong Tao, Weizhen Liu

**Affiliations:** 1 Department of Gastrointestinal Surgery, Union Hospital, Tongji Medical College, Huazhong University of Science and Technology, Wuhan, 430022 China; 2 Center for Biomolecular Science and Engineering, University of California, Santa Cruz, CA 95064 USA

**Keywords:** Metastasis, Gastric cancer

## Abstract

Tumor metastasis severely limits the prognosis of gastric cancer patients. RNA-binding proteins (RBPs) are crucial in tumor metastasis, yet there is limited research into their involvement in gastric cancer. Here, we found that ESRP1, a RBP specific in epithelial cells, is important in regulating the metastasis of gastric cancer cells. ESRP1 is negatively correlated with distant metastasis and lymph node metastasis in gastric cancer patients. And we demonstrated that ESRP1 inhibit migration and invasion of gastric cancer in vitro and in vivo. Mechanistically, ESRP1 promotes exon 11 alternative splicing of CLSTN1 pre-mRNA. The post-splicing short CLSTN1 stabilizes the Ecadherin/β-catenin binding structure, and promotes β-catenin protein ubiquitination and degradation, thereby inhibiting the migration and invasion of gastric cancer cells. Our study highlights the role of ESRP1 in regulating metastasis of gastric cancer and extends its mechanism. These results provide a possibility for ESRP1 and CLSTN1 to become therapeutic targets for metastasis of gastric cancer.

## Introduction

Gastric cancer is one of the most common malignant tumors, resulting in more than 1 million cases per year and 5.6% of all cancer diagnoses [1, 2]. Due to its hidden onset and the lack of specific clinical manifestations, gastric cancer patients are often diagnosed in the advanced stage, accompanied by local regional or distant metastasis [3]. Tumor metastasis is a major factor influencing the prognosis of gastric cancer, with the 5-year survival rate for early gastric cancer being as high as 70%, but less than 30% for advanced gastric cancer [4]. Thus, screening targets related to gastric cancer metastasis and exploring their function and the potential mechanisms can help to improve the treatment and prognosis of gastric cancer patients.

RNA binding proteins (RBPs) are a class of evolutionarily highly conserved proteins that possess RNA binding domains, which enable them to interact with RNA [5, 6]. RBPs comprise ~10% of all protein-coding genes [7]. They are involved in the alternative splicing regulation, transport, and localization of mRNA and play an important role in maintaining genomic diversity and stability [8–10]. Studies have demonstrated that RBPs are closely related to tumorigenesis and progression, and can influence tumor cell proliferation, epithelial-mesenchymal transition (EMT), and other processes by regulating pre-mRNAs alternative splicing [11–13].

Epithelial splicing regulatory protein 1 (ESRP1) is an RNA-binding protein and splicing factor specific to epithelial cells, which plays an important role in maintaining cell epithelial characteristics and regulating tumor progression [14, 15]. Currently, studies have shown that the role of ESRP1 is not consistent, it plays a role in promoting or suppressing cancer in different tumors. In pancreatic cancer, the ESRP1 expression is positively correlated with patient prognosis, and its overexpression significantly reduces liver metastasis [16]. In lung adenocarcinoma, ESRP1 inhibits tumor invasion and metastasis by regulating EMT [17]. On the other side, ESRP1 can act as an oncogene, regulating the biological functions of tumor cells and leading to lower overall survival of breast cancer patients and increased lung metastasis of 4T1 cells [18]. Also, the pro-metastasis function of ESRP1 were also proved in ovarian cancer and prostate cancer [19, 20]. In gastric cancer, previous study has shown that 85% of gastric cancer patients with low ESRP1 expression have disseminated gastric cancer [21]. This type of cancer is more prone to lymph node and peritoneal metastasis due to the lack of connexins between tumor cells, resulting in a poor prognosis. And ESRP1-induced isoform switching of LRRFIP2 may inhibit metastasis of gastric cancer [22].

In this study, we focused on the function of ESRP1 in gastric cancer and attempted to explore the regulation network of ESRP1. We demonstrated that expression of ESRP1 was negatively correlated with lymph node metastasis, distant metastasis, and TNM stage in gastric cancer patients. Further studies showed that ESRP1 protein can bind to CLSTN1 mRNA, mediating alternative splicing of exon 11. The post-splicing short CLSTN1 inhibits EMT of gastric cancer cells, and binds to β-catenin and stabilizing the cytoskeleton to inhibit invasion and migration of gastric cancer cells. Our results highlight the relationship between ESRP1 and gastric cancer metastasis, elucidating a novel CLSTN1-mediated invasion and metastasis mechanism. This emphasizes the potential of ESRP1 and CLSTN1 as attractive therapeutic targets in gastric cancer.

## Results

### ESRP1 is negatively correlated with gastric cancer metastasis

**Fig. 1:**
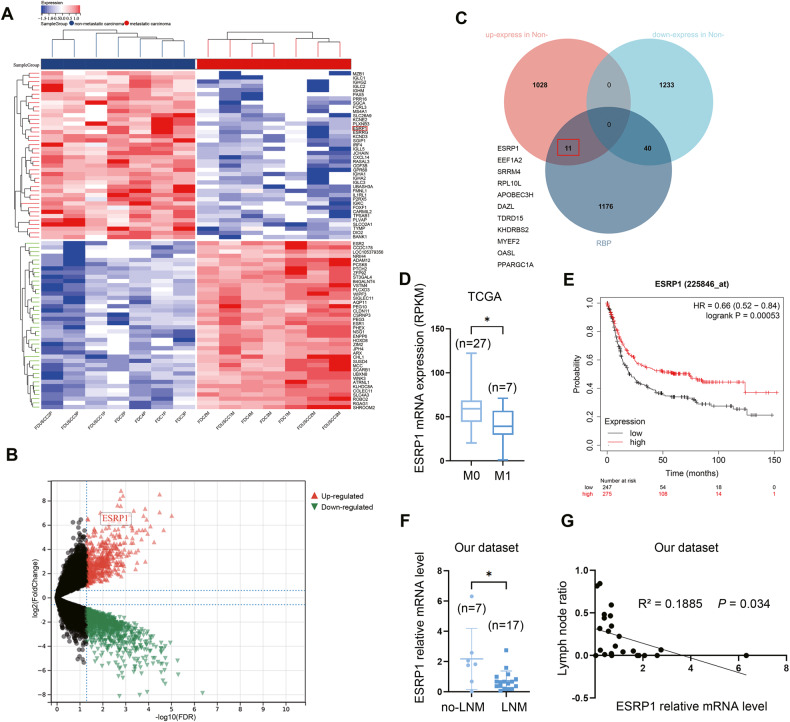
RNA binding protein ESRP1 is downregulated in metastatic gastric cancer. **A** Gene profile of the 2 datasets (GSE191139 and GSE206329) were analyzed according to primary tumor (non-metastatic) and metastatic tumor classification, the upregulated and downregulated top differential expression genes (DEGs) were shown in the heatmap. The color display corresponding expression abundance. **B** Volcano plot of upregulated and down-regulated DEGs, ESRP1 is labeled. **C** RNA binding proteins (RBPs) encoded by these DEGs were identified, the 11 RBPs upregulated in the non-metastatic gastric cancer were listed. **D** ESRP1 expression was compared between stomach adenocarcinoma patients with (M1) or without (M0) distant metastasis, using the TCGA data. Expression and metastasis data were downloaded from cBioPortal database. **E** Kaplan–Meier plot of gastric cancer patients. The patients were divided into 2 groups according to the expression of ESRP1 and compared with log-rank test. **F** ESRP1 expression (normalized to GAPDH) in gastric cancer surgical specimens from 24 patients were detected through qPCR, and the scatter plot shows difference of ESRP1 expression between metastatic and non-metastatic gastric cancer. LNM, lymph node metastasis; non-LNM, no lymph node metastasis. **G** The scattergram shows a linear relationship between ESRP1 expression and lymph node ratio (LNR), and the Pearson correlation coefficient was calculated. LNR was calculated by dividing the number of positive lymph nodes by the number of dissected lymph nodes. *P < 0.05.

We analyzed two public datasets (GSE191139 and GSE206329) and compared gene expression profiling of gastric cancer with or without metastasis. The top 80 differentially expressed genes (DEGs) (up and down) were visualized in the heatmap (Fig. 1A). We further filtered the DEGs according to their fold change and FDR (Fig. 1B). Of these DEGs, 40 RBPs were upregulated in metastatic gastric cancer, while 11 RBPs were highly expressed in non-metastatic gastric cancer, including ESRP1 (Fig. 1C). We then verified the relationship of ESRP1 expression and gastric cancer metastasis in TCGA database. Expression data and distant metastasis information of patients with stomach adenocarcinoma were download from cBioPortal. Patients with distant metastasis (M1, *n* = 7) show significant lower expression of ESRP1 than patients without distant metastasis (M0, *n* = 27) (Fig. 1D), indicating that ESRP1 is negatively correlated with tumor metastasis. And Kaplan–Meier analysis from the KM-plotter database show that patients with ESRP1 high expression have superior survival than those with low expression (HR = 0.66, 95% CI 0.52–0.84, *P* < 0.001) (Fig. 1E). Next, we further verified the role of ESRP1 in gastric cancer using our dataset. We collected 24 surgical specimens from gastric cancer patients and evaluated ESRP1 expression. We found that the expression of ESRP1 in gastric cancer patients without lymph node metastasis was significantly higher than in patients with lymph node metastasis (Fig. 1F). Further analysis revealed that ESRP1 expression was negatively correlated with lymph node metastasis rate (LNR), an important clinical indicator reflecting lymph node metastasis (Fig. 1G). These results all suggest that ESRP1 may act as a negative regulator of gastric cancer metastasis.

### ESRP1 inhibits the metastasis of gastric cancer cells in vitro and in vivo

**Fig. 2:**
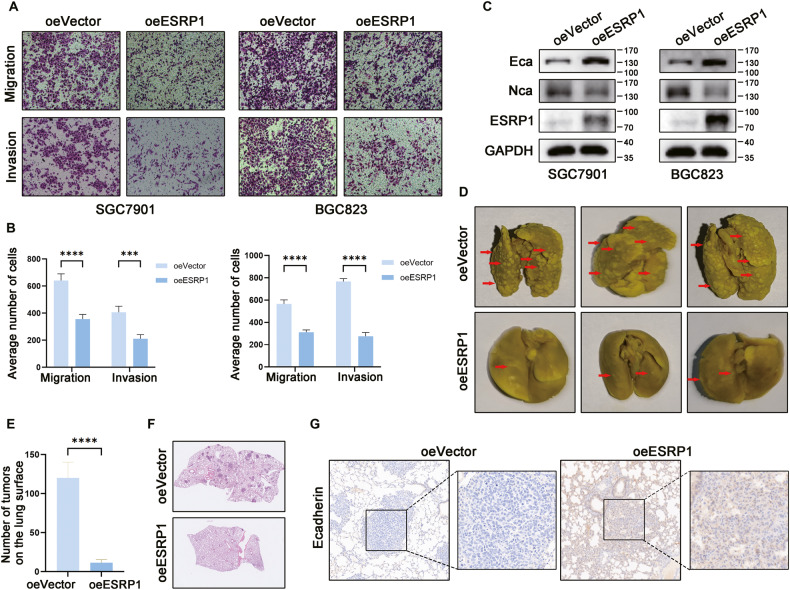
Overexpression of ESRP1 inhibits migration and invasion of gastric cancer in vitro and in vivo. **A** Transwell migration and invasion assay of control (oeVector) and ESRP1 overexpression (oeESRP1) gastric cancer cells. The left was SGC7901 gastric cancer cell and right was BGC823 cell. **B** Statistical histogram of the number of penetrated cells transwell migration and invasion assay. Left was SGC7901 and right was BGC823. **C** Western blot detecting the expression of Ecadherin, Ncadherin, ESRP1, and GAPDH protein in control (oeVector) and ESRP1 over expression (oeESRP1) gastric cancer cells. Left was SGC7901 cell and right was BGC823 cell. **D** In vivo analysis of ESRP1 overexpression influencing gastric cancer metastasis. 1 × 10^6^ oeVector or oeESRP1 SGC7901 cells were injected through tail vein (*n* = 5) and 1.5 months later, mice were sacrificed and lungs were dissected and fixed with Bouin’s Solution. The representative images were showed. Red arrows indicate lung metastases. **E** Number of metastases on the lung surface were counted and compared between oeVector and oeESRP1 groups. **F** Representative images of H&E staining of lungs in mice injected oeVector or oeESRP1 SGC7901 cells. **G** Representative images of IHC staining of Ecadherin in the lung metastases. Left was oeVector and right was oeESRP1 group. *P < 0.05, **P < 0.01, ***P < 0.001, ****P < 0.0001.

To further verify the influence of ESRP1 on metastasis of gastric cancer, we constructed two gastric cancer cell lines with overexpression of ESRP1 based on SGC7901 and BGC823, which have low endogenous ESRP1 expression levels. Transwell experiments showed that gastric cancer cells’ migration and invasion capacities decreased after overexpression of ESRP1 (Fig. 2A, B). Ecadherin and Ncadherin are two important indicators of epithelial-mesenchymal transition (EMT). We also found that Ecadherin expression increased while Ncadherin decreased after overexpression of ESRP1 in gastric cancer cells, suggesting that ESRP1 may inhibit the EMT process (Fig. 2C). We then verified the function of ESRP1 in vivo by mice tail vein injection assay. The results showed that the number of metastatic tumor nodules on the lungs of ESRP1-overexpressing group was significantly lower than that in the control group (Fig. 2D–F). Immunohistochemistry of mice lungs revealed that Ecadherin expression in lung metastases was significantly increased in the ESRP1 overexpression group (Fig. 2G).

**Fig. 3:**
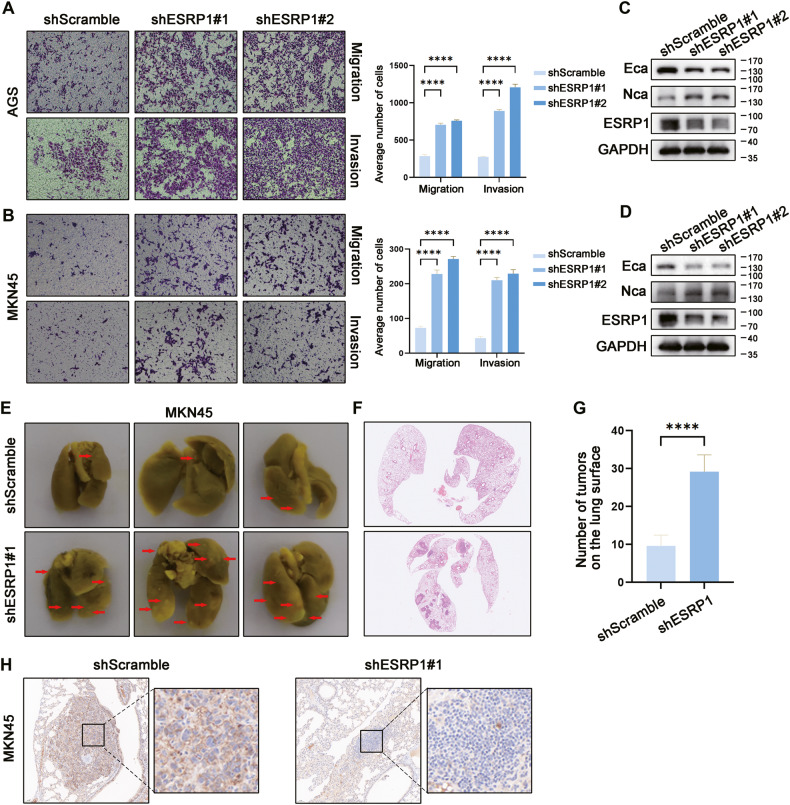
Knockdown ESRP1 promote migration and invasion of gastric cancer in vitro and in vivo. **A** Transwell migration and invasion assay of control (shScramble) and ESRP1 knockdown (shESRP1#1, shESPR1#2) AGS cells. Left were the representative images of transwell migration and invasion, right was the corresponding statistical histogram. **B** Transwell migration and invasion assay of MKN45 shScramble and shESRP1#1, shESRP1#2 cells. Left were the representative images and right was the corresponding statistical histogram. **C**, **D** Western blot analysis of Ecadherin, Ncadherin, ESRP1, and GAPDH protein in AGS (**C**) or MKN45 (**D**) cells with ESRP1 knockdown. **E** In vivo analysis of metastatic potential in MKN45 shScramble and shESRP1#1 cells. 1 × 10^6^ shScramble or shESRP1#1 MKN45 cells were injected through tail vein (*n* = 5) and 1.5 months later, mice were sacrificed and lungs were dissected and fixed with Bouin’s Solution. Representative images were displayed. **F** Representative images of lungs in which mice were injected with MKN45 shScramble or shESRP1#1 cells. **G** Statistical histogram of metastases number on the lung surface. **H** Representative images of IHC staining of Ecadherin in the lung metastases. Left was shScramble and right was shESRP1#1. ****P < 0.0001.

After ESRP1 knockdown, AGS cells and MKN45 cells showed increased migration and invasion in the transwell assay (Fig. 3A, B), and decreased Ecadherin expression and increased Ncadherin expression (Fig. 3C), suggesting that cells may undergo mesenchymal transition, which was further confirmed by the increased metastatic nodules in the lungs of mice injected with MKN45 shESRP1 cells (Fig. 3D–F) and lower levels of Ecadherin protein in the metastases (Fig. 3G). These results demonstrate that ESRP1 inhibits the metastasis of gastric cancer cells.

### ESRP1 regulate cell adhesion and cause a wide range of AS events

**Fig. 4:**
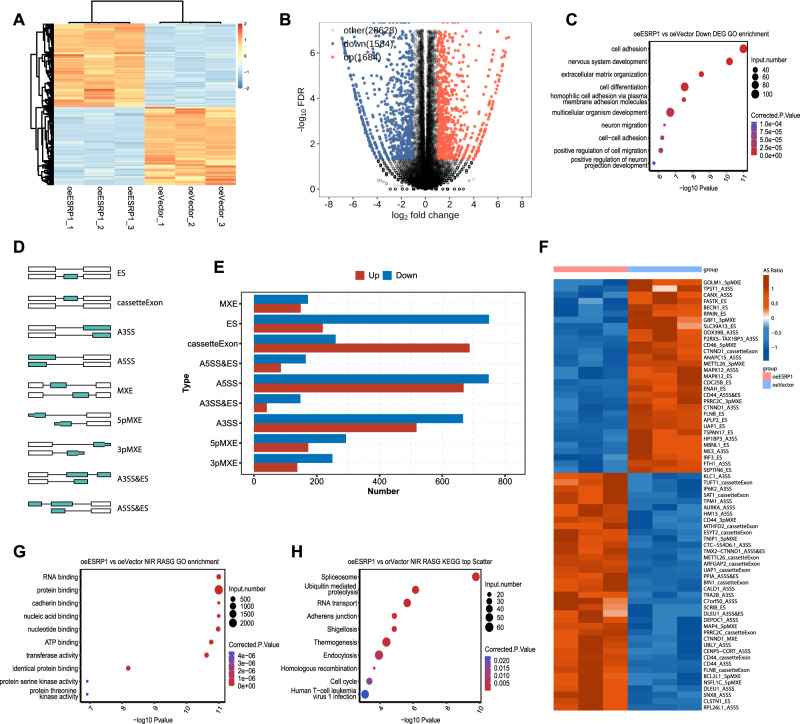
ESRP1 cause a wide range of AS events in gastric cancer cells. **A** Heatmap of DEGs between SGC7901 control (oeVector) and ESRP1 overexpression (oeESRP1) cells in RNA sequencing. **B** Volcano plot of upregulated and down-regulated DEGs in RNA sequencing. **C** Gene ontology (GO) enrichment analysis of down-regulated DEGs in oeESRP1 gastric cancer cells. **D** Schematic diagram of alternative splicing types. ES: exon skip. A3SS: alternative 3’ splice site. A5SS: alternative 5’ splice site. MXE: Mutually exclusive exons. 5pMXE: MXE + alternative 5’ promoter. 3pMXE: MXE + alternative polyadenylation site. **E** Histogram showing the number of different alternative splicing types. **F** Heatmap showing the top AS events in SGC7901 oeESRP1 gastric cancer cells. Top AS events were set as events with splicing counts > 50 and the difference AS ratio between groups > 0.2. Color of the ruler was displayed as AS ratio. **G** GO enrichment analysis of the differentially alternative splicing genes in SGC7901 oeESRP1 cells. **H** KEGG analysis of the differentially alternative splicing genes in SGC7901 oeESRP1 cells.

To further investigate the specific mechanism by which ESRP1 inhibits the metastasis of gastric cancer, we performed RNA sequencing (Fig. 4A) on SGC7901 cells with ESRP1 or Vector overexpression. A total of 3268 DEGs were identified, with 1684 genes upregulated and 1584 genes down-regulated in the overexpressing ESRP1 cells (Fig. 4B). GO enrichment analysis of the down-regulated DEGs were mainly related to cell adhesion (Fig. 4C), while the upregulated genes showed that they were mainly related to the extracellular region (Supplementary Fig. 1).

For the alternative splicing events, there are mainly nine types in mammalian cells, including exon skipping (ES), cassetteExon, A3SS, A5SS, MXE, etc. (Fig. 4D). Further analysis revealed that ESRP1-related alternative splicing events are mainly ES, A5SS, and A3SS, etc. (Fig. 4E). To quantitatively analyze the splicing of each gene induced by ESRP1, we calculated the alternative splicing ratio and the top AS events were presented in the heatmap (Fig. 4F). GO enrichment analysis of genes corresponding to differential AS events showed that these genes are closely related to RNA binding (Fig. 4G), while KEGG enrichment analysis indicated that these genes may be associated with spliceosome function (Fig. 4H). These results suggest that ESRP1 may regulate cell adhesion and cause a wide range of alternative splicing events in cells.

### ESRP1 binds to CLSTN1 exon 5 and promotes exon 11 skip splicing

**Fig. 5:**
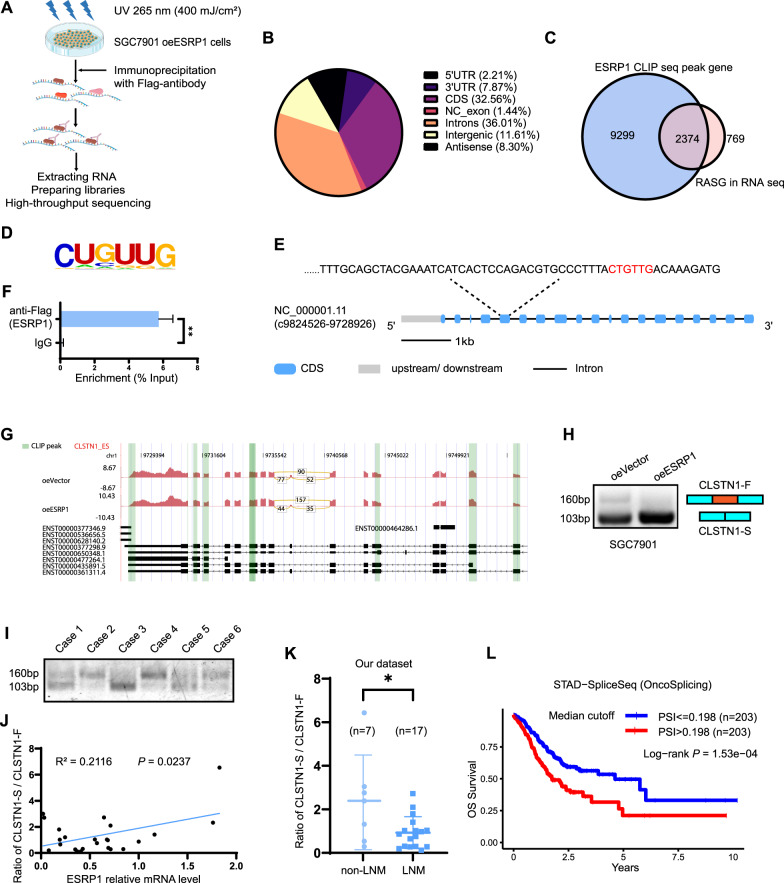
ESRP1 promote CLSTN1 exon 11 skip splicing. **A** Schema of the CLIP sequencing. Briefly, SGC7901 oeESRP1 gastric cancer cells were irradiated with UV once for 400 mJ/cm^2^, followed by lysis, immunoprecipitation with anti-Flag antibody, RNA extracting, library preparing, and high-throughput sequencing. **B** Pie chart of the distribution of ESRP1 binding sites. **C** Venn chart showing the shared genes in peak genes of CLIP seq and AS events related genes of RNA seq. **D** The motif of which ESRP1 binds to. **E** Schema of the CLSTN1 genome, and the possible binding sites were shown in red. **F** RIP PCR assay to verify that ESRP1 can interact with exon 5 region of CLSTN1 mRNA. The assay was conducted in SGC7901 oeESRP1 gastric cancer cells. **G** Schema of reads counts on CLSTN1 in RNA seq, and the CLIP peak were shown in green lines. **H** Representative images of agarose gel electrophoresis after PCR amplification of full-length (CLSTN1-F) and spliced CLSTN1 (CLSTN1-S) in SGC7901 oeVector and oeESRP1 gastric cancer cells. **I** Representative images of agarose gel electrophoresis after PCR amplification of CLSTN1-F and CLSTN1-S in gastric cancer specimens. **J** Scatter plot showing the correlation between CLSTN1 splicing ratio and ESRP1 expression in gastric cancer specimens (*n* = 24). CLSTN1 splicing ratio was calculated by gray release analysis of the agarose gel electrophoresis samples and ESRP1 expression was evaluated by qPCR. **K** Splicing ratio of CLSTN1 between gastric cancer patients with or without lymph node metastasis were compared. Non-LNM, no lymph node metastasis; LNM, lymph node metastasis. **L** Kaplan–Meier plot showed the relationship between CLSTN1 PSI score and prognosis in stomach adenocarcinoma patients in the TCGA database. The graph was drawn using SpliceSeq data from the OncoSplicing database. PSI, Percent spliced in. *P < 0.05.

Crosslinking and immunoprecipitation (CLIP and its variants) have been successfully applied to identify specific RNA-protein interactions in high resolution in both cell culture and living organisms or tissues. To identify ESRP1’s direct targets, we performed CLIP-seq in SGC7901 cells with ESRP1 overexpression (Fig. 5A). ESRP1 mainly binds to Introns (36.01%) and CDS (32.56%) (Fig. 5B). We identified a total of 9299 transcripts interacting with ESRP1 protein, of which 2374 had alternative splicing events (Fig. 5C). The top motif which ESRP1 binds to is ‘CUGUUG’ (Fig. 5D). Since RNA seq revealed that the AS ratio of SCRIB and CLSTN1 was significantly higher in SGC7901 cells with ESRP1 overexpression (Fig. 4F), we matched whether there were corresponding sites in the genome. In the CLSTN1 genome, there is a corresponding site in exon 5 (Fig. 5E). CLSTN1 belongs to the Calsyntenin family, a subset of the cadherin superfamily. We then confirmed that ESRP1 can bind to exon 5 of CLSTN1 by RIP PCR experiments (Fig. 5F). Joint analysis of CLIP-seq and RNA-seq showed that there were multiple peaks on CLSTN1 mRNA and ESRP1 mainly lead to the skip splicing of CLSTN1 exon 11 (Fig. 5G). We further designed primers across exon 11 for verification and found that, after overexpression of ESRP1, the truncated product (exon 11 skipping, 103 bp) of CLSTN1 increased, while the full length (exon 11 no skipping, 160 bp) decreased in gastric cancer cells (Fig. 5H). Subsequently, we verified this result on gastric cancer tissue specimens. Through PCR detection of 24 gastric cancer specimens, we found that the splicing ratio of CLSTN1 (CLSTN1-S/CLSTN1-F) was positively correlated with the expression level of ESRP1 (Fig. 5I, J). And splicing ratio of CLSTN1 in patients with lymph node metastasis (LNM) was significantly lower than that in the non-LNM patients (Fig. 5K). Survival analysis of the SpliceSeq and OncoSplicing database show that stomach adenocarcinoma patients with lower PSI (Percent spliced in) of CLSTN1 had a superior overall survival than patients with high PSI (Fig. 5L). These results indicated that ESRP1 could bind to exon 5 of CLSTN1 and induce skip splicing of exon 11, which was correlated with a low risk of lymph node metastasis and superior survival.

### CLSTN1 exon 11 splicing mediates ESRP1 induced inhibition of gastric cancer cells metastasis

**Fig. 6:**
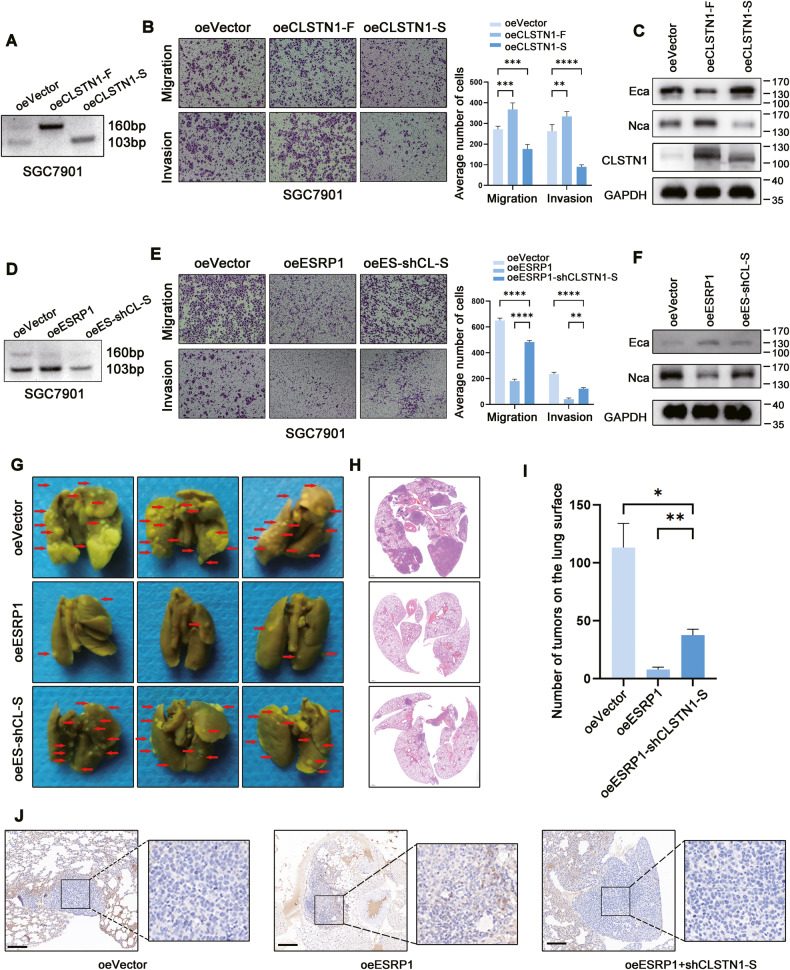
CLSTN1 exon 11 splicing mediates ESRP1 induced inhibition of gastric cancer cells metastasis. **A** Representative images of agarose gel electrophoresis after PCR amplification of CLSTN1 in control (oeVector), CLSTN1-F overexpression (oeCLSTN1-F), and CLSTN1-S overexpression (oeCLSTN1-S) SGC7901 gastric cancer cells. **B** Transwell migration and invasion assay in SGC7901 oeVector, oeCLSTN1-F, oeCLSTN1-S cells. Left were the representative images and right was the corresponding statistical histogram. **C** Representative images of western blot detecting the Ecadherin (Eca), Ncadherin (Nca), CLSTN1, and GAPDH (GA) protein in SGC7901 oeVector, oeCLSTN1-F, oeCLSTN1-S cells. **D** Representative images of agarose gel electrophoresis after PCR amplification of CLSTN1 in control (oeVector), oeESRP1, and oeESRP1 plus CLSTN1-S knockdown (oeES-shCL-S) SGC7901 gastric cancer cells. **E** Transwell migration and invasion assay in SGC7901 oeVector, oeESRP1, oeES-shCL-S cells. Left were the representative images and right was the statistical histogram. **F** Representative images of western blot detecting the Ecadherin (Eca), Ncadherin (Nca), and GAPDH protein in SGC7901 oeVector, oeESRP1, oeES-shCL-S cells. **G** Metastases on the lung surface of mice injected with SGC7901 oeVector, oeESRP1, or oeES-shCL-S cells through tail vein (*n* = 5). Lungs were fixed with Bouin’s Solution and representative images were displayed. Red arrows indicate lung metastases. **H** Representative images of H&E staining of lungs in the three groups of mice. Top, oeVector; middle, oeESRP1; down, oeES-shCL-S. **I** Statistical histogram of metastases number on the lung surface. **J** Representative images of IHC staining of Ecadherin in the lung metastases. Left, oeVector; middle, oeESRP1; right, oeES-shCL-S. ns, no significance. *P < 0.05, **P < 0.01, ***P < 0.001, ****P < 0.0001.

To study the role of CLSTN1 in gastric cancer metastasis, we constructed full-length (CLSTN1-F) and truncated (CLSTN1-S) overexpression plasmids (Fig. 6A). Through the transwell assay, we observed that overexpression of the truncated CLSTN1 (CLSTN1-S) significantly reduced cell migration and invasion, while overexpression of the CLSTN1-F promoted migration and invasion of gastric cancer cells (Fig. 6B). In addition, we examined the effect of CLSTN1 on key EMT proteins and found that CLSTN1-S promoted Ecadherin protein expression and inhibited Ncadherin expression, while CLSTN1-F had the opposite effect (Fig. 6C), suggesting that CLSTN1-S may mediates ESRP1 induced inhibition of gastric cancer metastasis. To further test this hypothesis, we knocked down CLSTN1-S (oeES-shCL-S) (Fig. 6D) in gastric cancer cells overexpressing ESRP1. The rescue assay showed that knocking down CLSTN1-S significantly reversed the ESRP1-induced reduction in migration and invasion of gastric cancer cells in the transwell assay (Fig. 6E). Furthermore, cells in the oeES-shCL-S group exhibited lower levels of Ecadherin and higher levels of Ncadherin compared to the oeESRP1 group (Fig. 6F), indicating that knockdown of CLSTN1-S might induce EMT in ESRP1 overexpression cells. We then further validated this phenomenon in mice, wherein the indicated three groups of cells were injected into the tail vein, and lung metastatic nodules were examined one month later. We found that knocking down CLSTN1-S significantly reversed the reduction in lung metastasis caused by overexpression of ESRP1 (Fig. 6G–I). We also performed immunohistochemical staining of Ecadherin in mouse lungs, and, as before, overexpression of ESRP1 promoted Ecadherin expression on tumor cells; however, knocking down CLSTN1-S impaired this effect (Fig. 6J). These results suggest that CLSTN1-S plays a critical role in mediating ESRP1 to inhibit metastasis of gastric cancer cells.

### CLSTN1 affect the stability of Ecadherin/β-catenin adhesive structure

**Fig. 7:**
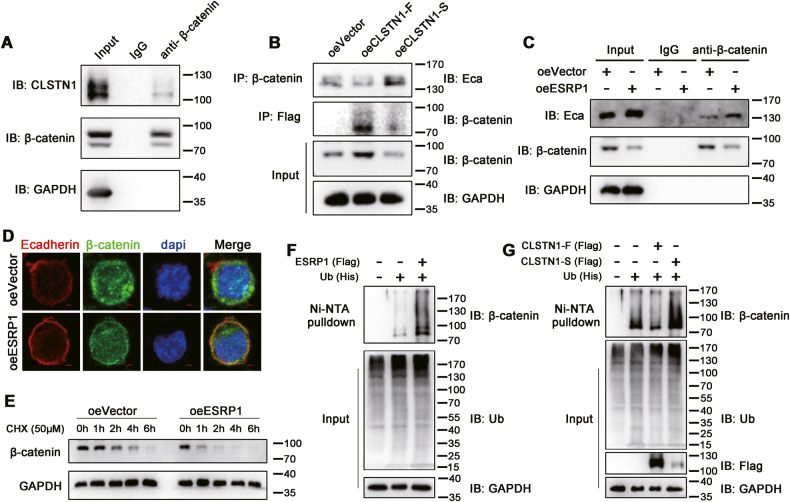
CLSTN1 affect the interaction of Ecadherin and β-catenin structure. **A** Co-immunoprecipitation of CLSTN1 and β-catenin protein in SGC7901 gastric cancer cells. IB: immune blotting. **B** co-immunoprecipitation of β-catenin and Ecadherin, CLSTN1-F, CLSTN1-S in SGC7901 oeVector, oeCLSTN1-F, and oeCLSTN1-S cells. IP: immunoprecipitation. **C** co-immunoprecipitation of β-catenin and Ecadherin in SGC7901 oeESRP1 gastric cancer cells. **D** Immunofluorescence co-localization of β-catenin and Ecadherin in SGC7901 oeVector or oeESRP1 cells. Representative images were shown. **E** CHX assay of SGC7901 gastric cancer cells oeVector or oeESRP1. **F** Ni-NTA pulldown of HEK293T cells transfected with ESRP1 and/or ubiquitin plasmids. ESRP1 (Flag), Flag-tagged ESRP1 plasmid; Ub (His), His-tagged ubiquitin plasmid. **G** Ni-NTA pulldown of HEK293T cells transfected with CLSTN1-F, CLSTN1-S, and/or ubiquitin plasmids. CLSTN1-F (Flag), Flag-tagged CLSTN1-F plasmid; CLSTN1-S (Flag), Flag-tagged CLSTN1-S plasmid.

The interaction of Ecadherin to β-catenin plays an important role in maintaining cytoskeleton stability and reducing cell invasion and metastasis. To further investigate whether CLSTN1, a cadherin, is involved in this process, we conducted co-IP experiment and found that endogenous CLSTN1 and β-catenin interact in gastric cancer cells (Fig. 7A). We then overexpressed CLSTN1-F or CLSTN1-S in gastric cancer cells to detect whether the truncated and full length CLSTN1 have different affinities to β-catenin. We found that CLSTN1-F could promote β-catenin expression, while CLSTN1-S down-regulated β-catenin protein. In addition, our results show that after overexpression of CLSTN1-F, the interaction of β-catenin and Ecadherin was reduced, while this interaction was increased when CLSTN1-S was overexpressed (Fig. 7B). Moreover, an increased interaction of β-catenin and Ecadherin was also observed in gastric cancer cells overexpressing ESRP1. Consistently, overexpression of ESRP1 lead to decreased β-catenin protein in SGC7901 gastric cancer cells (Fig. 7C). This finding was further verified in the immunofluorescence experiment. As shown in Fig. 7D, ESRP1 overexpression led to a reduction of β-catenin fluorescence, while resulted in an increase of colocalization of β-catenin and Ecadherin on the cell membrane. Since β-catenin is usually degraded in a ubiquitin-proteasome manner, we further explored whether ESRP1 promotes the degradation of β-catenin. Through the CHX assay, we found that the degradation rate of β-catenin after overexpression of ESRP1 was higher than that of the control group (Fig. 7E). Furthermore, our results demonstrated that ESRP1 and CLSTN1-S could promote ubiquitination of β-catenin, while CLSTN1-F inhibited this process (Fig. 7F, G). Taken together, these results suggested that, on one hand, CLSTN1-S promoted the interaction of Ecadherin and β-catenin to maintain cytoskeleton stability and cell adhesion; on the other hand, CLSTN1-S could promote the ubiquitination and degradation of β-catenin. These may be the mechanisms by which ESRP1 inhibits gastric cancer metastasis through alternative splicing of CLSTN1.

## Discussion

Gastric cancer is one of the most common malignancies worldwide, with the fourth highest mortality rate, largely due to tumor metastasis [1]. Alternative splicing is an important source of protein diversity and has been shown to regulate various biological functions, including invasion and migration [23–25]. In this study, we confirmed that ESRP1, an RNA-binding protein, can inhibit epithelial-mesenchymal transition and metastasis of gastric cancer by promoting alternative splicing of exon 11 of CLSTN1 mRNA. The functions of ESRP1 and CLSTN1 as biomarkers have also been verified in clinical samples, providing the potential for screening individuals at risk of tumor metastasis and early clinical intervention.

RBP is an important class of proteins in cells. Through its special RNA binding domain, it is widely involved in various post-transcriptional regulatory processes such as RNA splicing, localization, degradation, editing, modification and translation [26–28]. ESRP1 is a well-known RBP associated with tumor metastasis. And previous studies indicated that ESRP1 might play a completely different role in reproductive system tumors and digestive system tumors. In gastric cancer, it has been reported that ESRP1 can promote exon 7 splicing of LRRFIP2 and inhibit migration and invasion [22]. In this study, we further explored the mechanism of ESRP1 inhibiting metastasis of gastric cancer through CLIP sequencing. Our results show that ESRP1-regulated CLSTN1 exon 11 alternative splicing also plays an important role in the invasion and metastasis of gastric cancer.

Calsyntenin-1 (CLSTN1), a type I transmembrane protein belonging to the cadherin superfamily, is a unique family of calcium-binding proteins that links extracellular proteolytic activity to intracellular Ca^2+^ signaling [29]. CLSTN1 is mainly expressed in brain nerve cells and has been poorly studied in tumors [30, 31]. Its biological processes include cell adhesion and proliferation, and an N-linked glycosylation checkpoint in aspartate 515 in its alternative splicing exon 11 suggests that the two transcripts CLSTN1-F and CLSTN1-S may have different biological functions. We found that the exon 11 spliced transcript (CLSTN1-S) can inhibit invasion and migration of gastric cancer cells, and targeting CLSTN1-S can reverse ESRP1-induced invasion and migration inhibition. This is consistent with the previous study reported that splice isoform switching of CLSTN1 is crucial for EMT in breast cancer [32].

Tumor metastasis is a complex cascade process, which is closely related to several biological process such as EMT, extracellular matrix changes, and angiogenesis [33–35]. EMT is a complex process with a primordial role in cellular transformation whereby an epithelial cell transforms and acquires a mesenchymal phenotype, and is accompanied with a decrease in the expression of the cell epithelial marker Ecadherin and an increase in the expression of the mesenchymal marker Ncadherin [35, 36]. It is regulated by multiple signaling pathways, with the Wnt/β-catenin pathway playing an important role [37]. β-catenin is an intracellular scaffold protein that interacts with Ecadherin at cell junction and participates in the formation of adhesive bands [38, 39]. Once triggered by Wnt ligands, the β-catenin protein is stabilized and accumulated in the cytoplasm and further translocates into the nucleus where β-catenin initiate downstream target gene expression, including matrix metallopeptidase-7 (MMP-7) [40, 41]. Our results demonstrate that ESRP1-mediated CLSTN1 exon 11 splicing can increase Ecadherin expression, thus inhibiting EMT in gastric cancer cells. Furthermore, CLSTN1-S overexpression may enhance the interaction between β-catenin and Ecadherin, indicating that the structure of Ecadherin adhesive bands on the cell membrane is more stable. These may be key mechanisms by which ESRP1 and CLSTN1-S suppress metastasis of gastric cancer cells.

This study also has some limitations. The effects of ESRP1 and CLSTN1-S on β-catenin transposition and activation were not studied, and the specific mechanism of CLSTN1-S in promoting Ecadherin expression and inhibiting Ncadherin expression has not been further analyzed, and further research is needed. Nevertheless, our study provides new evidence and insights for ESRP1 to inhibit invasion and migration of gastric cancer, thus enriching its mechanism.

In conclusion, our study confirmed that ESRP1 expression and CLSTN1 splicing level were negatively correlated with metastasis of gastric cancer in vitro, in vivo, and in clinical samples. We found that ESRP1 drives CLSTN1 exon 11 splicing, resulting in the spliced CLSTN1 (short CLSTN1), which participates in inhibiting tumor cell EMT and stabilizing Ecadherin/β-catenin adhesive structure, which is an important mechanism for inhibiting gastric cancer metastasis. Our study provides new insights into the molecular regulation of ESRP1-CLSTN1 and its role in gastric cancer metastasis. A deeper understanding of the molecular regulation of ESRP1-CLSTN1 will facilitate the design of new strategies to treat or prevent metastasis of gastric cancer.

## Materials and methods

### Cells and gastric cancer tissue

Human gastric cancer cell lines SGC7901, BGC823, AGS, and MKN45 were purchased from the National Collection of Authenticated Cell Cultures of China. And these cells were cultured with RPMI-1640 medium (Gibco) supplemented with 10% fetal bovine serum (FBS, Gibco). Human embryonic kidney cell line HEK293T was obtained from the National Collection of Authenticated Cell Cultures of China and cultured with Dulbecco’s Modifed Eagle’s Medium with high glucose, 10% FBS. All these cells were cultured in moist incubator with 5% CO_2_ at 37 °C.

Gastric cancer surgical specimens of 24 consecutive patients were obtained from the Department of Gastrointestinal Surgery of Union Hospital, Tongji Medical College, Huazhong University of Science and Technology. Written informed consent of all the patients were obtained and the study was approved by the Ethics Committee of Union Hospital, Tongji Medical College, Huazhong University of Science and Technology, and complied with the Helsinki Declaration (approval no. IEC-J-349).

### Lentivirus and transfection

Lentivirus for ESRP1, CLSTN1-F (full length), CLSTN1-S (truncated) overexpression and ESRP1, CLSTN1-S knockdown were purchased from Genechem (Shanghai). Sequence of the short hairpin RNAs were shown in Supplementary Table 1. Stable cell lines with the indicated genes overexpression or/and knockdown were generated by transfection and screening with puromycin, according to the manufacturer’s instructions.

### Transwell migration and invasion assay

For migration assay, exuberant cells were harvested and resuspension in RPMI-1640 medium (Gibco) supplemented with 2% FBS. 400 μL suspension medium containing 1.2 × 10^5^ cells were added into the transwell chamber (Corning), and 500 μL culture medium with 10% FBS was added into the lower chamber of the 24-wells plate. After cultured for 18 h, the transwell chamber was fixed with 4% paraformaldehyde for 20 min, followed by stained with crystal violet for 30 min. Then transwell chamber was gently washed and air-dried. Cells on the membrane was counted with a microscope.

For invasion assay, Matrigel matrix (#354248) was used to simulate the extracellular matrix environment. Briefly, Matrigel matrix that thawed at 4 °C overnight was diluted with pre-cold opti-MEM (Gibco) at the ratio of 1:3, 165 μL diluted Matrigel matrix was added into the transwell chamber and placed in the incubator at 37 °C for 30 min. The unbounded Matrigel matrix was gently removed. Exuberant cells were harvested and resuspended to the concentration of 1.5 × 10^5^ cells per 400 μL using RPMI-1640 medium containing 2% FBS. 400 μL suspension medium was added into the chamber and the lower compartment was filled with 500 μL medium containing 10% FBS. After cultured at 37 °C for 24 h, cells on the membrane of the transwell chamber was fixed, stained and then observed and counted under the microscope.

### Western blot and immunoprecipitation

Whole cell protein was extracted using the RIPA lysate (#R0278, Sigma) containing protease inhibitor cocktail (#B14001, Biomake) and the concentration was measured by the BCA kit (#P0012, Beyotime). Protein was boiled for 10 min after added the loading buffer (#P0015, Beyotime). Then the protein was subjected to sodium dodecyl sulfate–polyacrylamide gel electrophoresis (SDS-PAGE) and transferred to polyvinylidene fuoride membrane (Millipore). After blocked with 5% skim milk for 30 min at room temperature, the membrane was incubated with indicated primary antibody at 4 °C overnight. Then the membrane was washed and incubated with corresponding secondary antibody conjugated with horseradish peroxidase (HRP) at room temperature for 1 h. The membranes were subjected to chemiluminescence imaging with ECL reagents (#6883, Cell Signaling Technology) using the Invitrogen iBright CL1500 imaging system (Thermo Fisher Scientific). The primary antibodies used in the western blot included anti-ESRP1 (21045-1-AP, Proteintech, 1:1000), anti-Ecadherin (#14472, Cell Signaling Technology, 1:1000), anti-Ncadherin (#13116, Cell Signaling Technology, 1:1000), anti-CLSTN1 (12788-1-AP, Proteintech, 1:1000), anti-GAPDH (#60004–1-Ig, Proteintech, 1:3000), anti-βcatenin (#A19657, Abclonal, 1:1000). The secondary antibodies included HRP conjugated goat anti rabbit IgG H&L (#511203, ZENBIO, 1:3000) and goat anti mouse IgG H&L (#511103, ZENBIO, 1:3000).

For immunoprecipitation (IP), cells were harvested and total protein were extracted. After measure the protein concentration, primary antibody was added into the lysate and incubated on the rotary shaker at 4 °C overnight. Then protein A/G magnetic beads or anti-Flag immunomagnetic beads were added and continue incubation for 4 h at 4 °C. The beads were washed and boiled with loading buffer and then subjected to SDS-PAGE. The primary antibodies used in the IP assay included anti-βcatenin (#A19657, Abclonal, 1:300). And the homologous rabbit IgG (#A7016, Beyotime) was used as a control.

### Ni-NTA pulldown

The ubiquitination of β-catenin was detected by Ni-NTA pulldown assay. Briefly, His-tagged ubiquitin plasmid and Flag-tagged ESRP1, CLSTN1-F, and CLSTN1-S plasmid were constructed. The indicated plasmids were co-transfected into HEK293T cells. Two days later the cells were treated with MG132 (50 μM) for 4 h and then harvested. Cells were washed with PBS and then lysed using urea lysis buffer (8 M urea, 0.1 M NaH_2_PO_4_, 300 mM NaCl, and 0.01 M pH8.0 Tris Hcl). The lysates were ultrasonicated briefly until it became fluid. Parts of the lysates were taken as input and the remaining were incubated with Ni-NTA agarose beads (HY-K0210, MCE) on rotary shaker for 2 h at room temperature. Then, beads were washed in urea lysis buffer for 5 times, loading buffer was added and boiled for 10 min. The pulldown products and input were subjected to SDS-PAGE detection.

### PCR and agarose gel electrophoresis

Total RNA in gastric cancer cells or specimens were extracted using RNAiso Plus* (#9109, Takara) and reverse transcribed using PrimeScript RT Master Mix (#RR036A, Takara). The ESRP1 mRNA level was detected by quantitative real-time PCR using TB Green Premix Ex Taq II (#RR820A, Takara) on a StepOne Plus System (Termo Fisher Scientifc). And comparative Ct method (2^−ΔΔCt^) was used to calculate the standardized expression level of ESRP1 mRNA.

To verify the two transcripts of CLSTN1 (CLSTN1-F and CLSTN1-S), a primer spanning exon 11 was designed. RNA from gastric cancer specimens were reverse transcribed and amplified with the primer by Takara TP600 PCR system (Takara). The products were subjected to agarose gel electrophoresis and the bands were imaging with TS-GelRed nucleic acid gel dye (TSJ003, Tsingke) using the Invitrogen iBright CL1500 imaging system (Termo Fisher Scientifc). Determine the gray release value of the band by ImageJ and calculate the ratio of the two transcripts as the splicing ratio. And correlation of the CLSTN1 splicing ratio and ESRP1 mRNA level, lymph node metastasis, lymph node ratio (LNR, calculated as positive lymph nodes number divided by dissected lymph nodes number) were analyzed.

### Tail vein injection assay in mice

Tail vein injection assay was conducted to identify the metastatic potential of gastric cancer cells in vivo. Four-week-old balb/c null mice were purchased from HUAFUKANG Bioscience. Mice were randomized divided into each group and every group had 5 mice (n = 5). SGC7901 (1.0 × 10^6^) or MKN45 (1.5 × 10^6^) cells in 100 μL phosphate-buffered saline (PBS) were injected in mice through the tail vein. One and a half months later, mice were euthanized and the lungs were dissected. Lungs were fixed with Bouin’s Fixative Solution at 4 °C overnight. After dehydrated with 70% alcohol, the lungs were embedded in paraffin. The double-blind method was adopted in the animal experiment, and the groups of mice were not clear for the tail vein injectors and the investigator who sacrificed the mice. The in vivo assays were approved by the Ethics Committee of Union Hospital, Tongji Medical College, Huazhong University of Science and Technology and complied with the Institutional Animal Care and Use Committee (IACUC) guidelines (approval no. 3256).

### Histology and Immunohistochemistry (IHC) staining

The embedded lungs were subjected to prepare sections of 5–6 mm thickness. Hematoxylin and eosin (H&E) staining followed routine methods was conducted to confirm the histological diagnosis of lung metastasis and other microscopic characteristics. For IHC staining, the lung sections were deparaffinized with xylene and rehydrated with gradient alcohol. After blocking the activity of endogenous peroxidase and antigen repair, the sections were subjected to block the non-specific antibody binding sites with 5% bovine serum albumin. Then, sections were incubated with anti-Ecadherin (#14472, Cell Signaling Technology, 1:200) antibody at 37 °C for 1 h, followed by incubated with secondary antibody, and subsequently stained with the SABC kit (#SA1054, Boster Biological Technology) according to the manufacturer’s instructions.

### RNA sequencing

For transcripts detection, SGC7901 cell lines with Vector or ESRP1 overexpression were constructed. RNA of 1 × 10^7^ cells were extracted and RNA sequencing was conducted by BGI company.

### Crosslinking-immunprecipitation (CLIP) sequencing

The CLIP seq was conducted by the Ablife institute (Wuhan). Briefly, SGC7901 cells were irradiated once for 400 mJ/cm^2^, and lysed in ice-cold wash buffer (1× PBS, 0.1% SDS, 0.5% NP-40, and 0.5% sodium deoxycholate) supplemented with a 200 U/mL RNase inhibitor (Takara) and protease inhibitor cocktail (Roche) and incubate on ice for 30 min. After centrifuge, RQ I (Promega) with a final concentration of 1 U/μL was added to the supernatant and incubate for 30 min at 37 °C. Immediately afterward, a stop solution was added to the lysates to quench DNase. The mixture was then vibrated vigorously and centrifuged at 13,000 × *g* for 20 min at 4 °C to remove cell debris. Then RNA digestion by MNase (Thermo Fisher Scientific) was performed.

For immunoprecipitation, the supernatant was incubated overnight at 4 °C with 10 μg Flag-antibody (#14793, Cell Signaling Technology) and control IgG-antibody (#2729, Cell Signaling Technology). The immunuprecipitates were further incubated with protein A/G Dynabeads (Thermo Scientific) for 2 h at 4 °C. After applying to magnet and removing the supernatants, the beads were sequentially washed with lysis buffer, high-salt buffer (250 mM Tris 7.4, 750 mM NaCl, 10 mM EDTA, 0.1% SDS, 0.5% NP-40, and 0.5 deoxycholate), and PNK buffer (50 mM Tris, 20 mM EGTA and 0.5% NP-40) twice, respectively. Resuspend the beads with elution buffer (50 nM Tris 8.0, 10 mM EDTA, and 1% SDS). Incubate the suspension for 20 min in a heat block at 70 °C to release the immunoprecipitated RBP with crosslinked RNA and vortex. Remove the magnetic beads and add Proteinase K (Roche) into the 1% input (without immunoprecipitated) and immunoprecipitated RBP with crosslinked RNA, with final concentration of 1.2 mg/ml. Incubate for 120 min at 55 °C. The RNA was purified with RNAiso Plus* (#9109, Takara). cDNA libraries were prepared with the KAPA RNA Hyper Prep Kit (#KK8541, KAPA) according to the manufacturer’s procedure. For high-throughput sequencing, the libraries were prepared following the manufacturer’s instructions and applied to Illumina NovaSeq system for 150 nt paired-end sequencing.

### RNA immunoprecipitation (RIP) PCR

RIP was conducted as previously described with minor modification [42, 43]. Briefly, 3 × 10^7^ SGC7901 cells with ESRP1 overexpression were harvested and washed with PBS twice. Cells were lysed with polysome lysis buffer containing protease inhibitor cocktail and RNase inhibitor. After removing the DNA, 10% of the lysate was taken out as input, and the remaining was incubated with ESRP1 antibody on rotary shaker overnight at 4 °C. Then, protein A/G magnetic beads were added and keep on incubating for 2 h. The beads were washed with wash buffer for 5 times and the binding RNA were extracted with RNAiso Plus* (#9109, Takara) and reverse into cDNA using PrimeScript RT Master Mix (#RR036A, Takara). The CLSTN1 mRNA region to which ESRP1 binds was verified by PCR experiments.

### Bioinformatic analysis

To study the RBPs that involved in gastric cancer metastasis, two datasets of paired primary gastric cancer, paracancerous tissues, and the metastasis (GSE191139 and GSE206329) were downloaded from the Gene Expression Omnibus (GEO) database. (https://www.ncbi.nlm.nih.gov/geo/). The “affy” package in R4.0.0 was used for standardization and transformation of the datasets, the “Limma” package was used to analyze the differentially expressed genes (DEGs) between primary cancer and distant metastatic cancer, and the visualization was performed by “pheatmap” package. Venn plots were used to analyze and visualize RBPs involved in the DEGs. Correlation of ESRP1 expression and distant metastasis (M stage) in patients with stomach adenocarcinoma from the Cancer Genome Atlas Program (TCGA) database was analysis through cBioPortal [44]. The cutoff value of Zscore was set as 1.2. Effect of ESRP1 expression on progression-free survival in gastric cancer patients was analyzed by Kaplan–Meier Plotter [45], the log-rank test was used to compare differences in survival between the two groups of patients. The relationship between alternative splicing of CLSTN1 exon 11 and prognosis in gastric cancer patients was analyzed using SpliceSeq [46] data from the OncoSplicing database [47].

### Statistical analysis

GraphPad Prism 9.0 (GraphPad Software, Inc., La Jolla, CA) were used for statistical analysis. Results were shown in mean ± standard deviation (SD). Student’s t-test was applied to compare mean between two normally distributed data, while Mann–Whitney test was used for comparison of differences in non-normally distributed cases. And one way ANOVA was used to analyze the differences between multiple groups. Pearson correlation coefficient was used to analyze the relationship between ESRP1, CLSTN1 splicing and metastasis. Statistical significance was set at two-tailed *P* < 0.05.

### Supplementary information

supplementary figure1

supplementary table1

supplementary material

## Data Availability

Both the RNA-seq data (accession number GSE233685) and the CLIP-seq data (accession number GSE233931) are deposited at NCBI Gene Expression Omnibus. Original data of the western blot were shown in the supplementary material.
