# Supplementary material for: ESRP1-driven alternative splicing of CLSTN1 inhibits the metastasis of gastric cancer
Source: Cell Death Discov. 2023 Dec 19;9:464. doi: 10.1038/s41420-023-01757-8 (PMC10730894; doi:10.1038/s41420-023-01757-8)

**Supplementary Figure1. ESRP1 is related to extracellular region.** Go enrichment analysis of the up-regulated genes in ESRP1 overexpression cells.


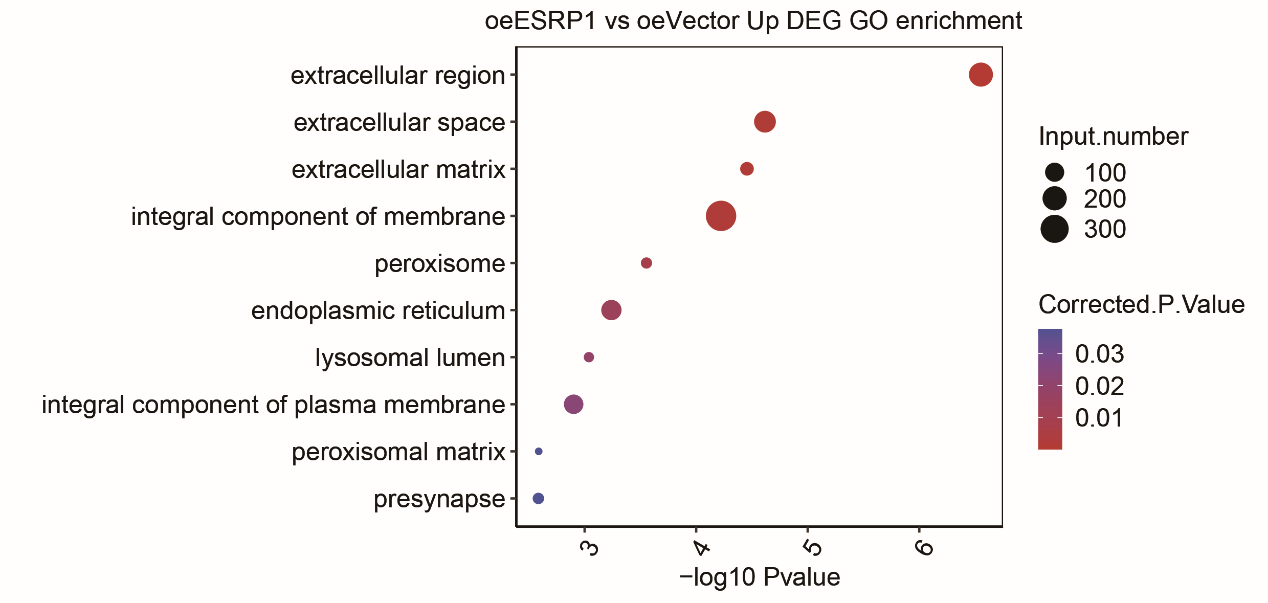

Supplement: Supplementary file 1 — supplementary figure1 [file 41420_2023_1757_MOESM1_ESM.docx]
