# Supplementary material for: ESRP1-driven alternative splicing of CLSTN1 inhibits the metastasis of gastric cancer
Source: Cell Death Discov. 2023 Dec 19;9:464. doi: 10.1038/s41420-023-01757-8 (PMC10730894; doi:10.1038/s41420-023-01757-8)
Supplement: Supplementary file 3 — supplementary material [file 41420_2023_1757_MOESM3_ESM.pdf]

Full and uncropped western blots images

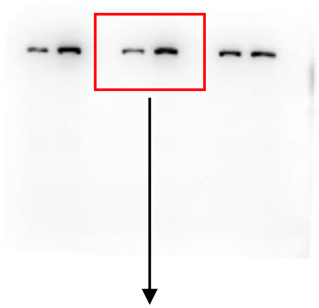

Fig2C. SGC7901 Eca

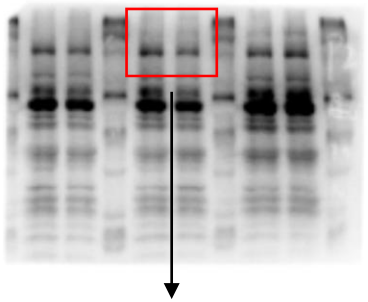

Fig2C. SGC7901 Nca

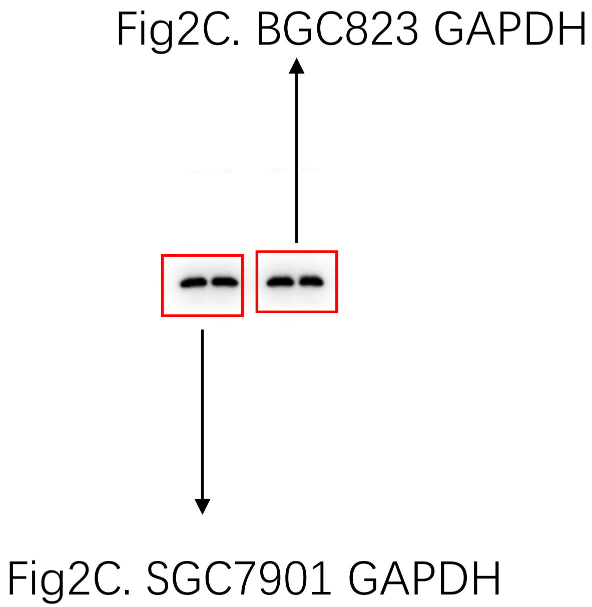
Fig2C. BGC823 GAPDH
Fig2C. SGC7901 GAPDH

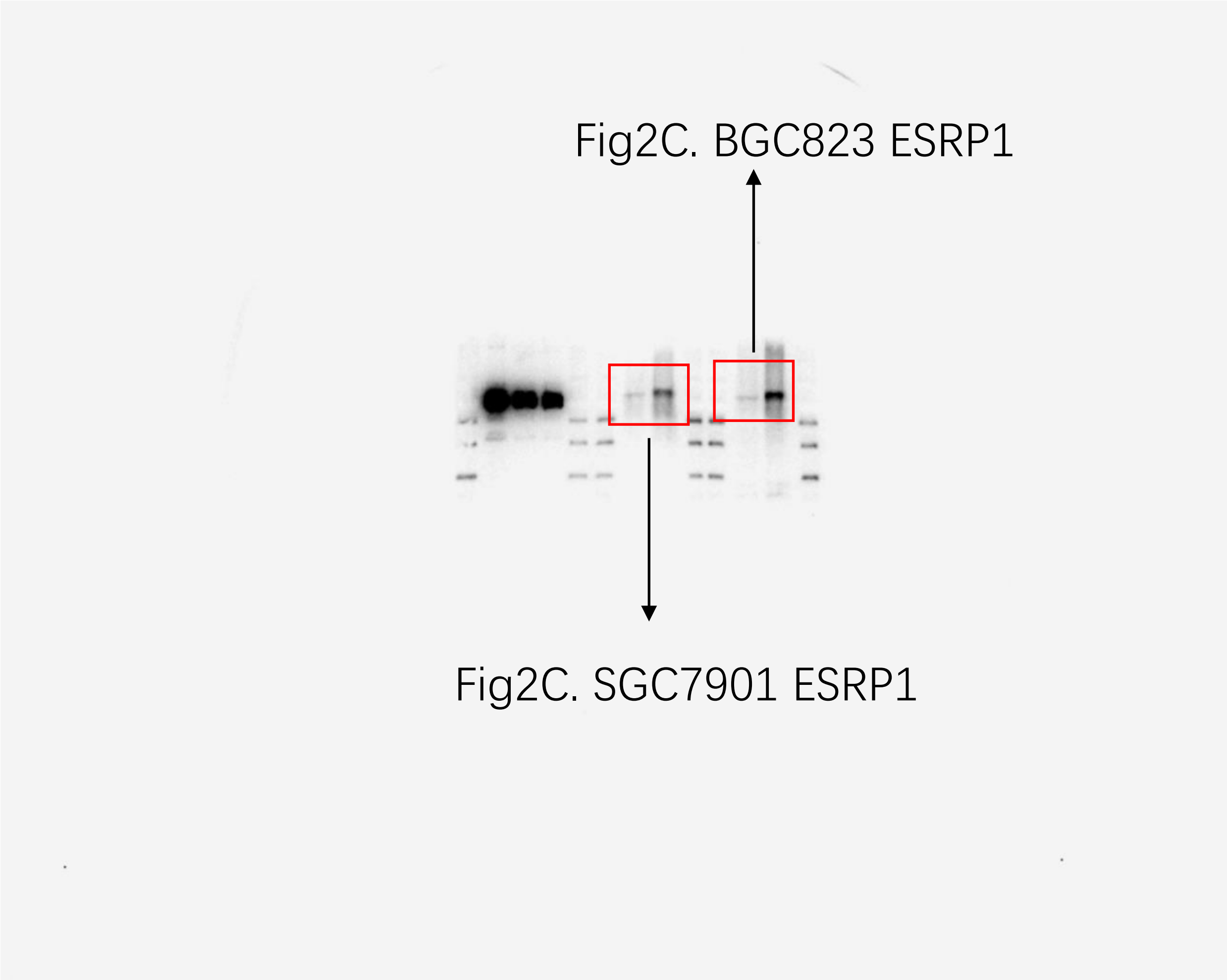
Fig2C. BGC823 ESRP1
Fig2C. SGC7901 ESRP1

Fig2C. BGC823 Eca

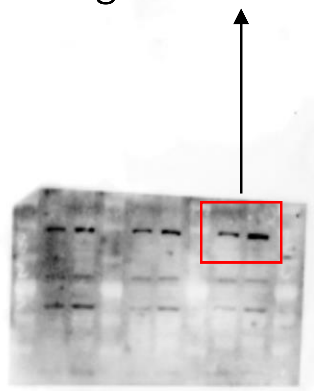

Fig2C. BGC823 Nca

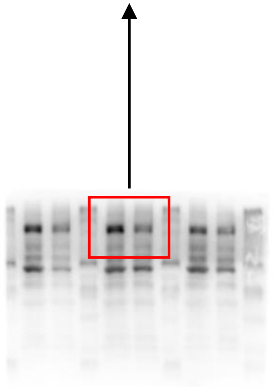

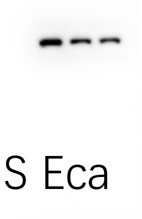

Fig3C. AGS Eca

Fig3C. AGS Nca

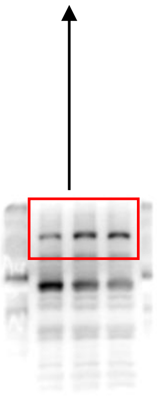

Fig3C. AGS ESRP1

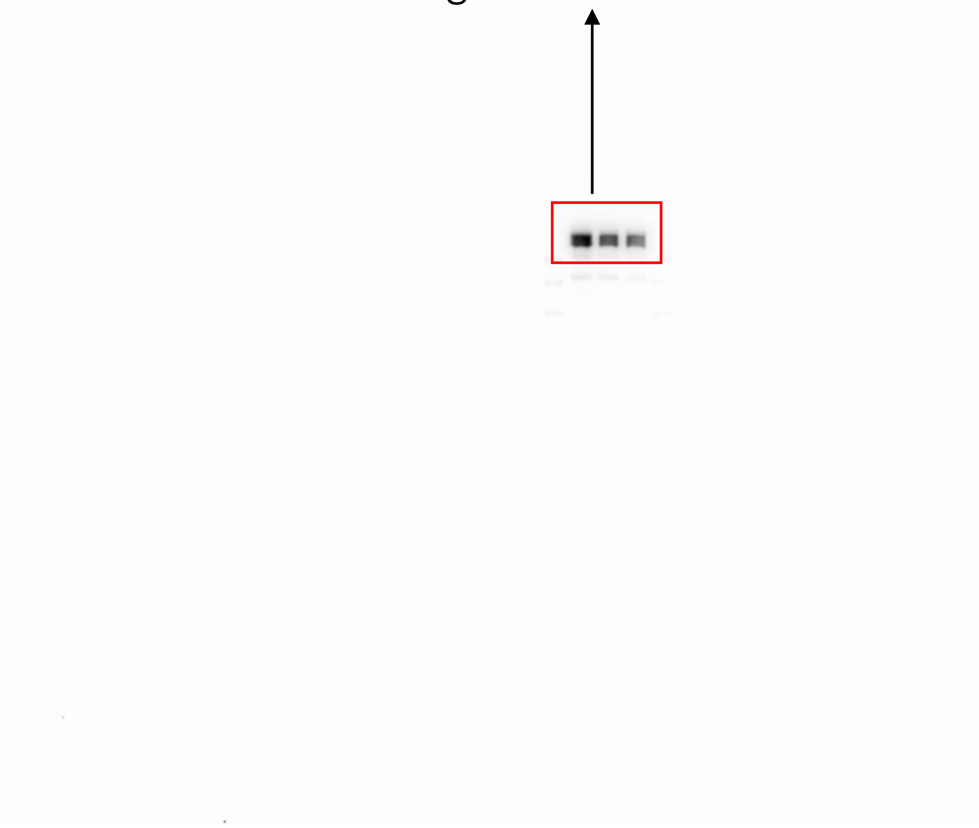

Fig3C. MKN45 Eca

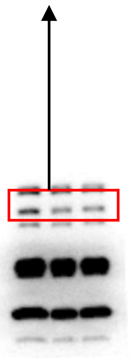

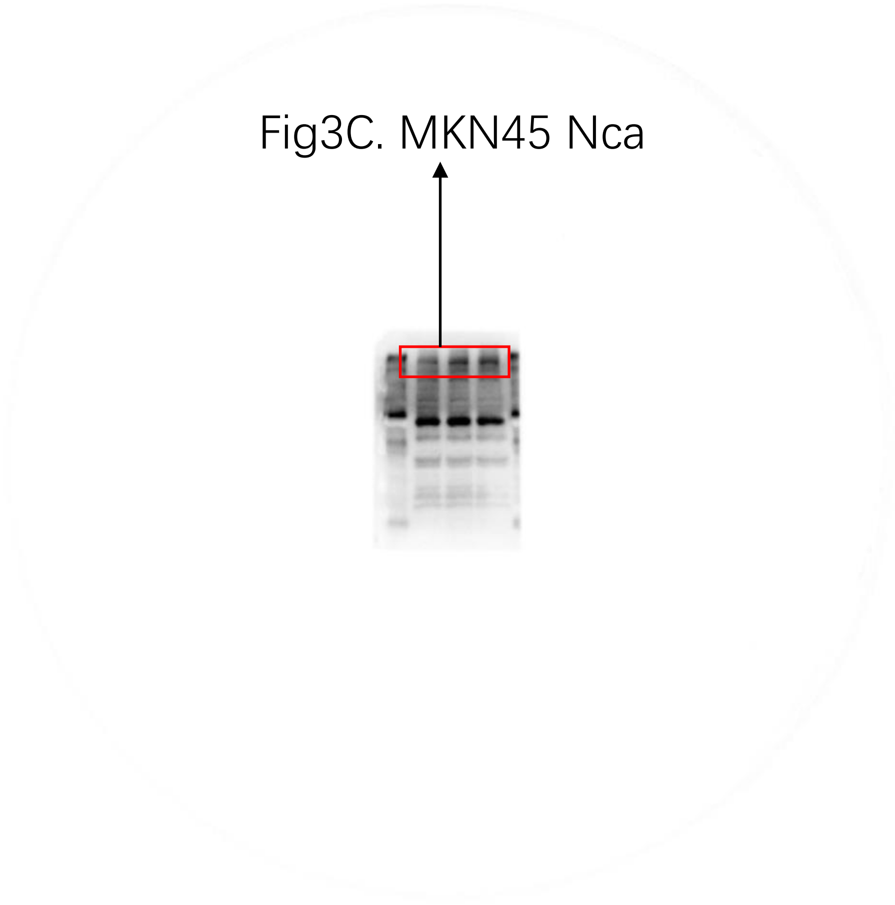
Fig3C. MKN45 Nca

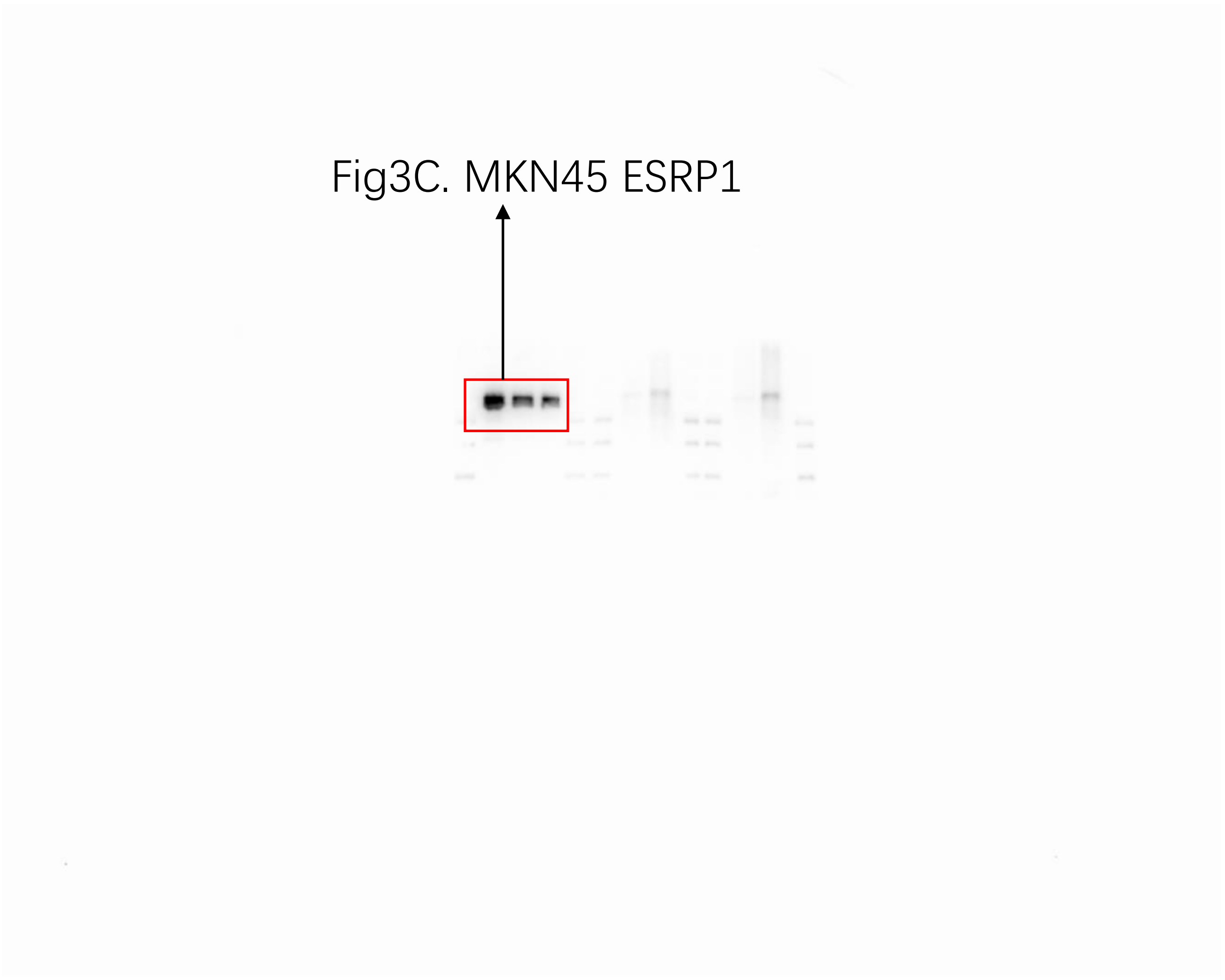
Fig3C. MKN45 ESRP1

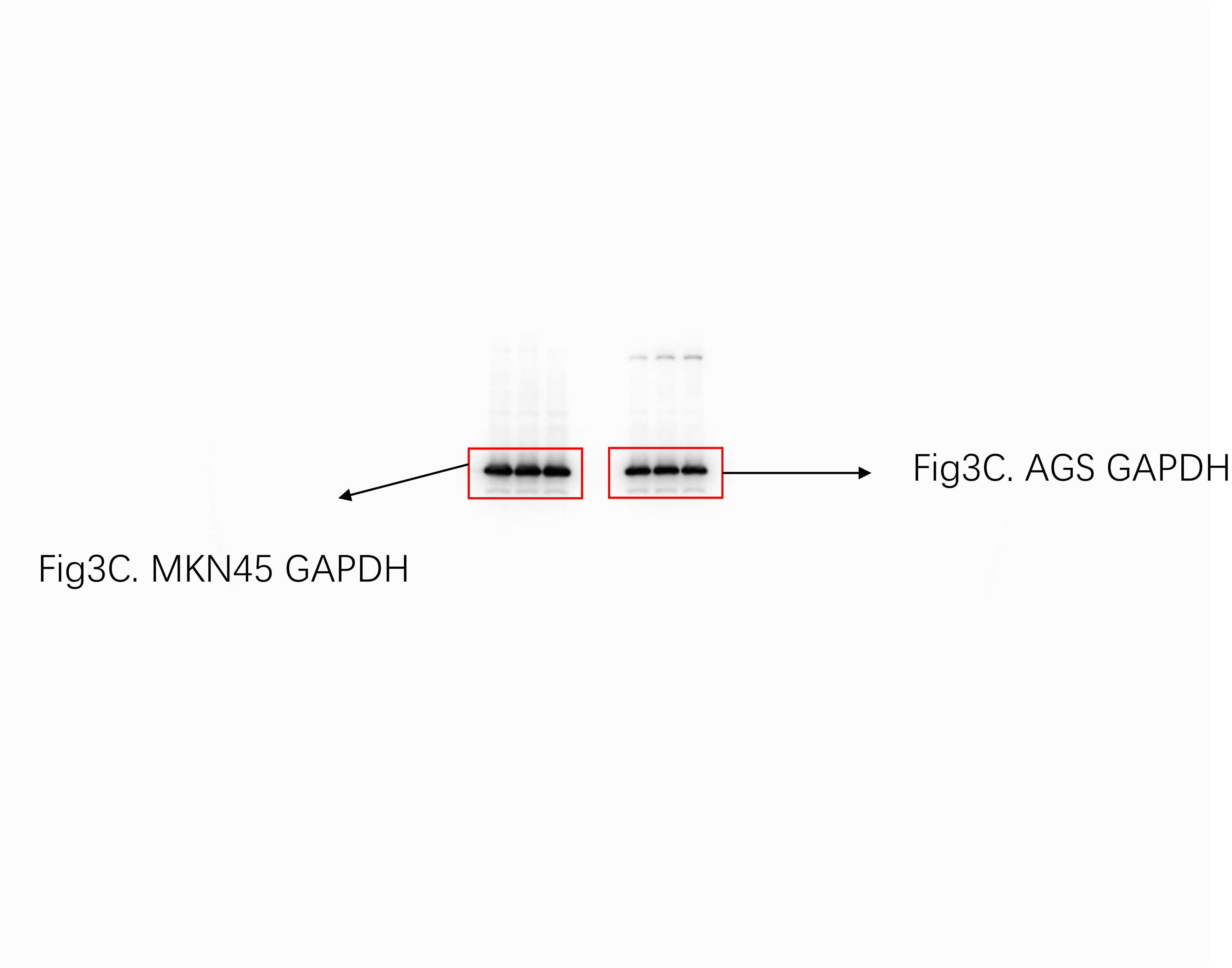
Fig3C. AGS GAPDH
Fig3C. MKN45 GAPDH

Full and uncropped nucleic acid images

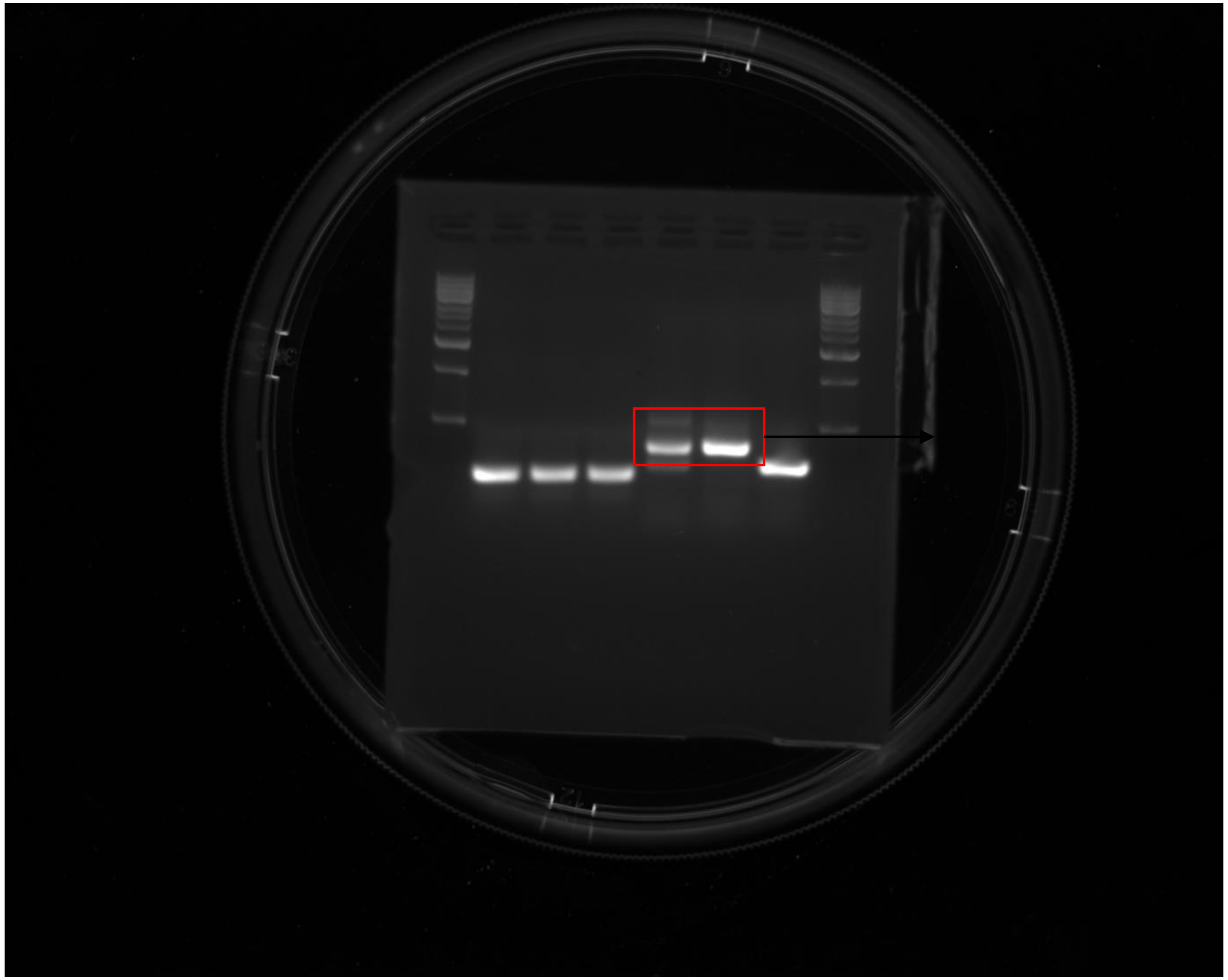

Fig5H

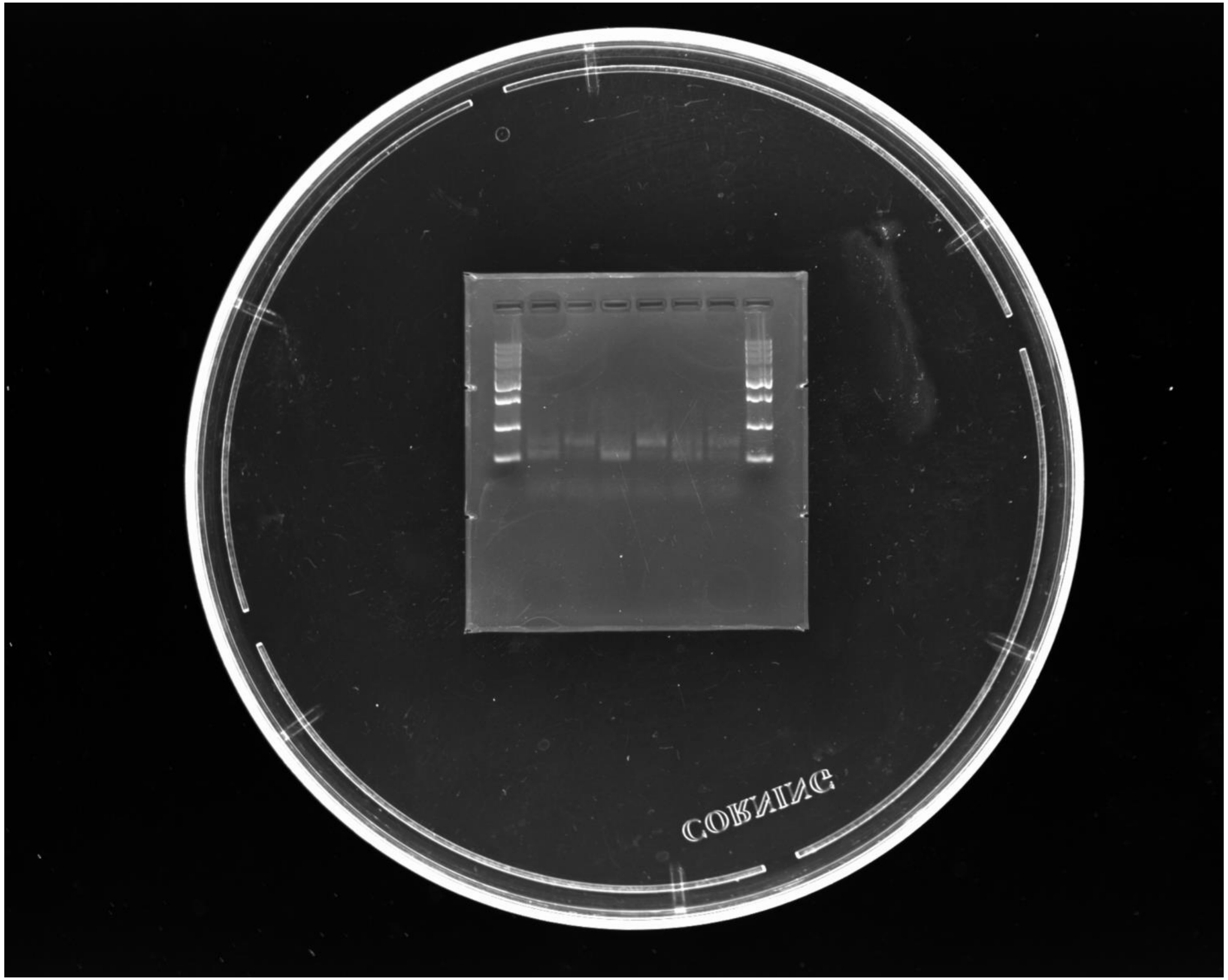


Fig5I

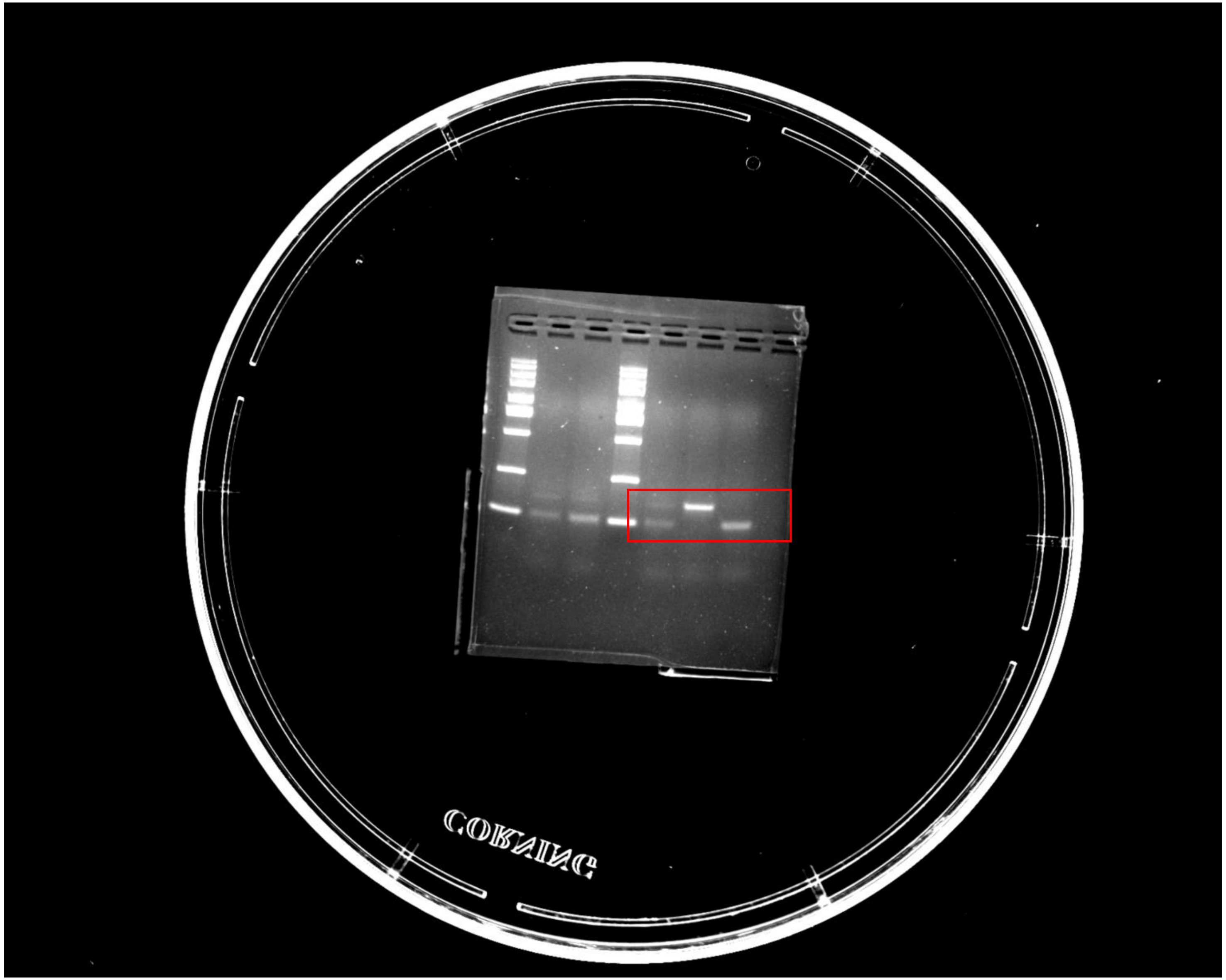

Fig6A

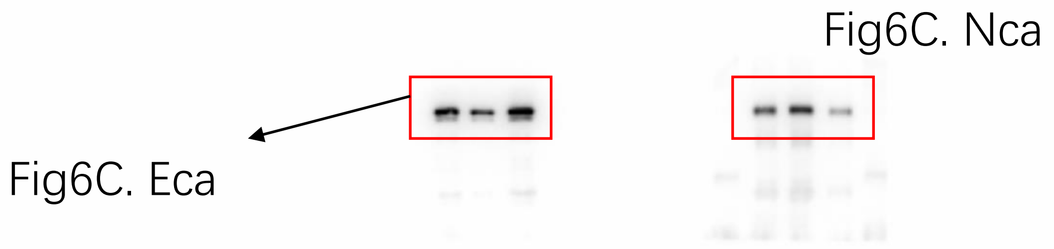
Fig6C. Nca
Fig6C. Eca

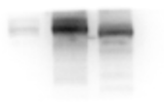

Fig6C. CLSTN1

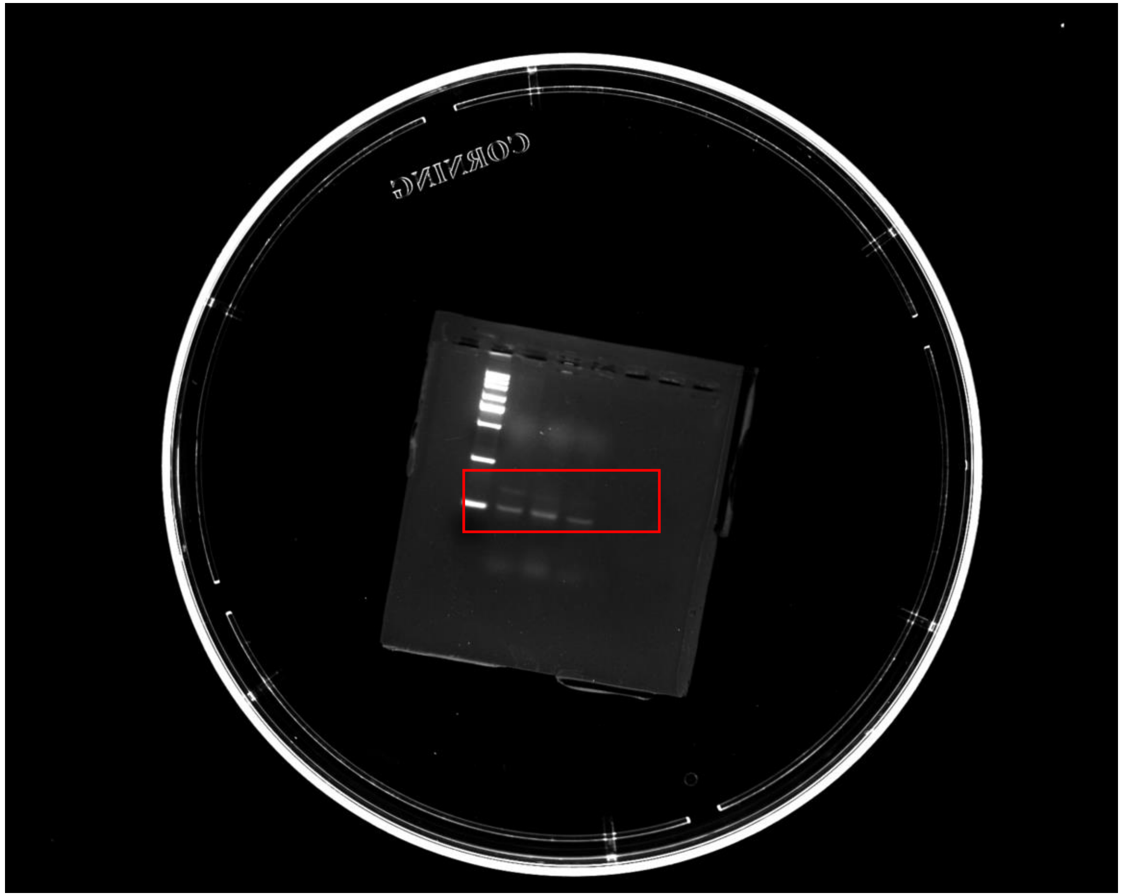

Fig6D

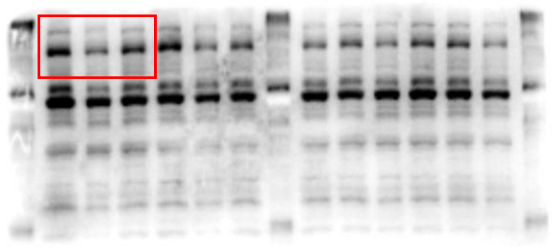

Fig6F Nca

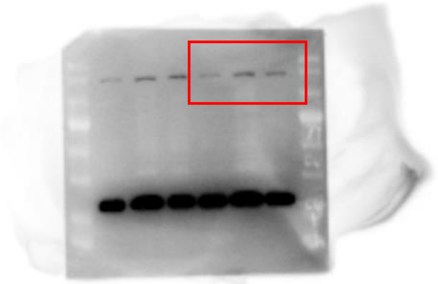

Fig6F Eca

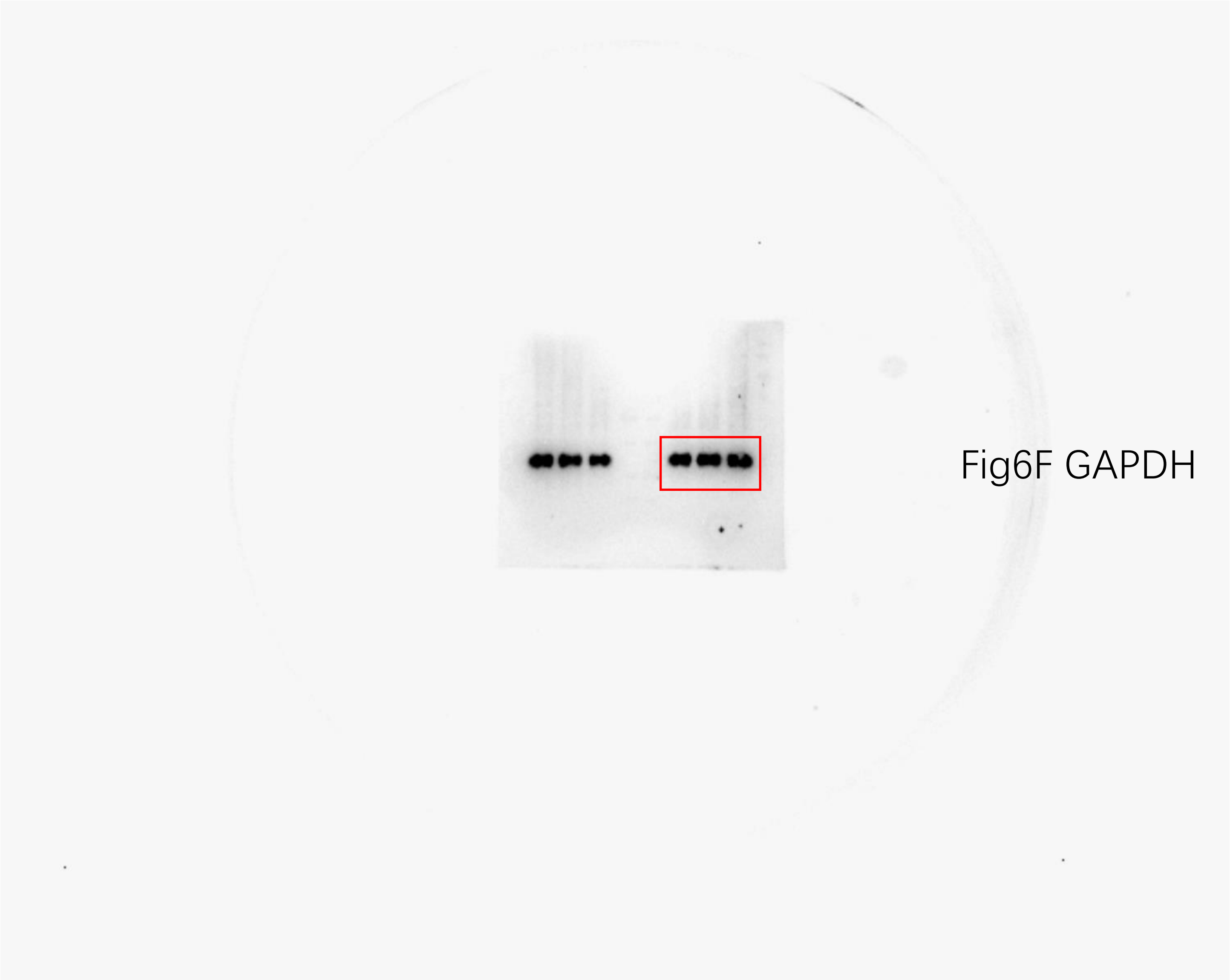

Fig6F GAPDH

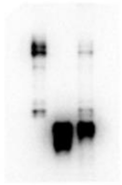

Fig7A IB:CLSTN1

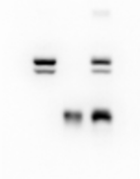

Fig7A IB:β-catenin

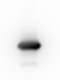

Fig7A IB:GAPDH

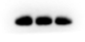

Fig7B GAPDH

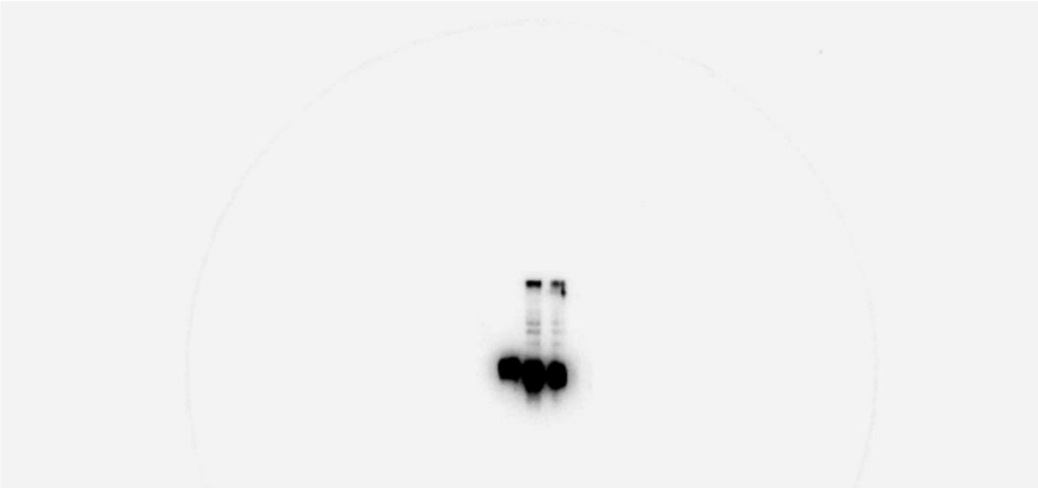

Fig7B IP:flag IB: b-catenin

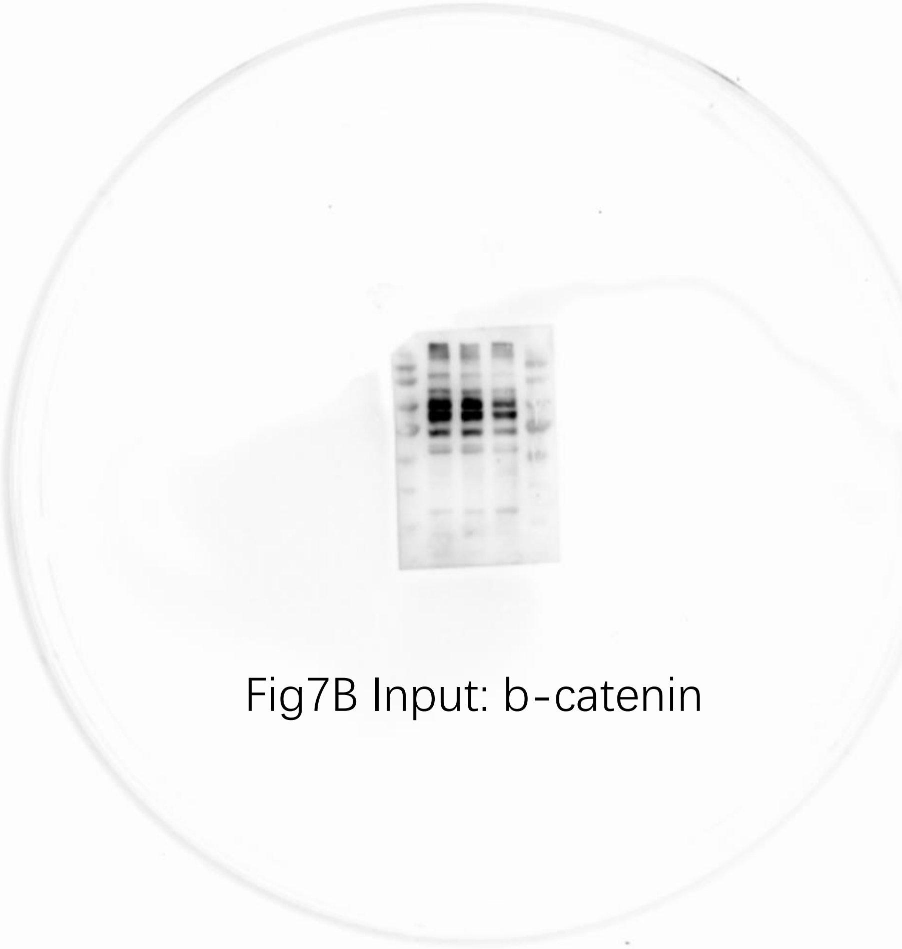

Fig7B Input: b-catenin

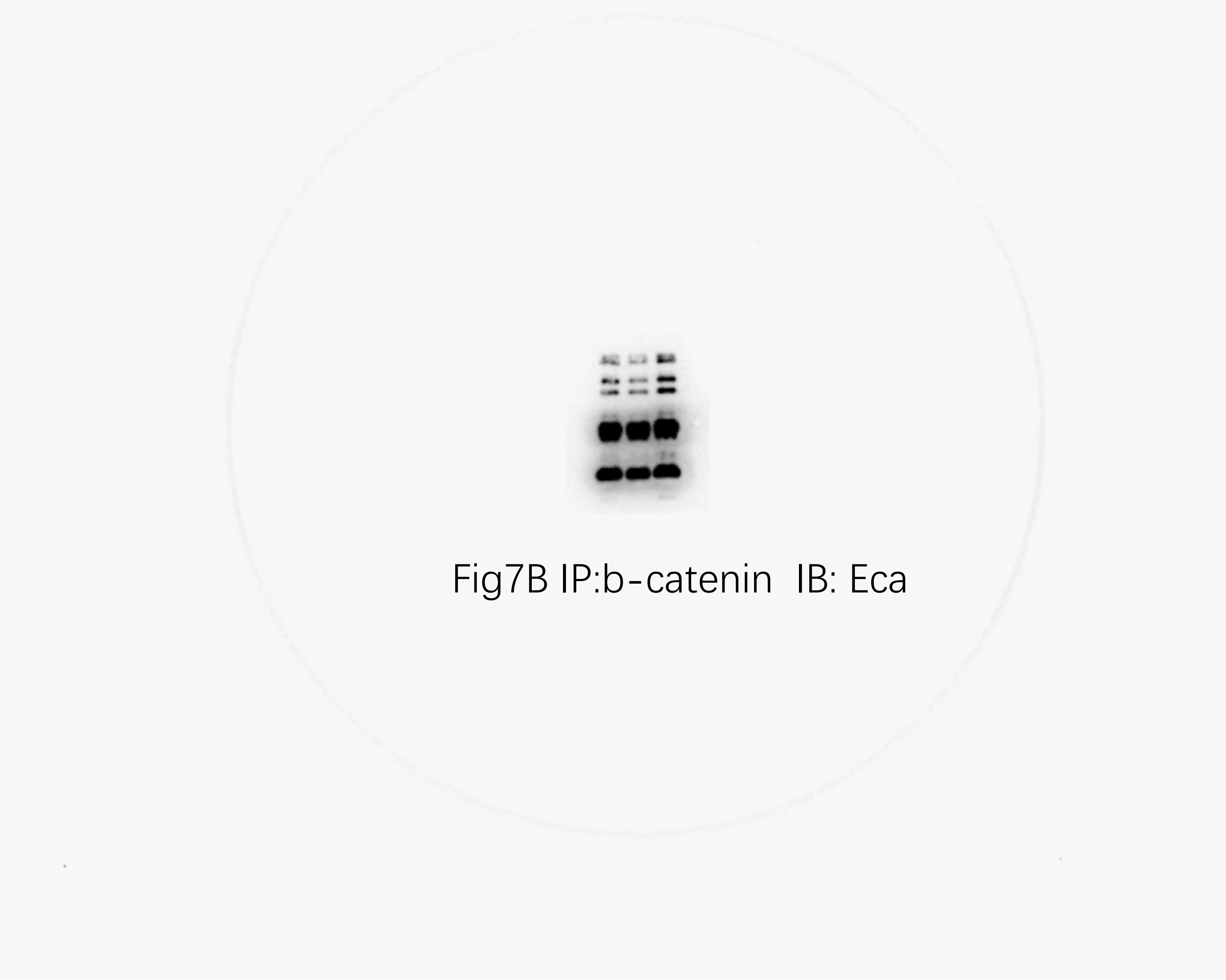

Fig7B IP:b-catenin  IB: Eca

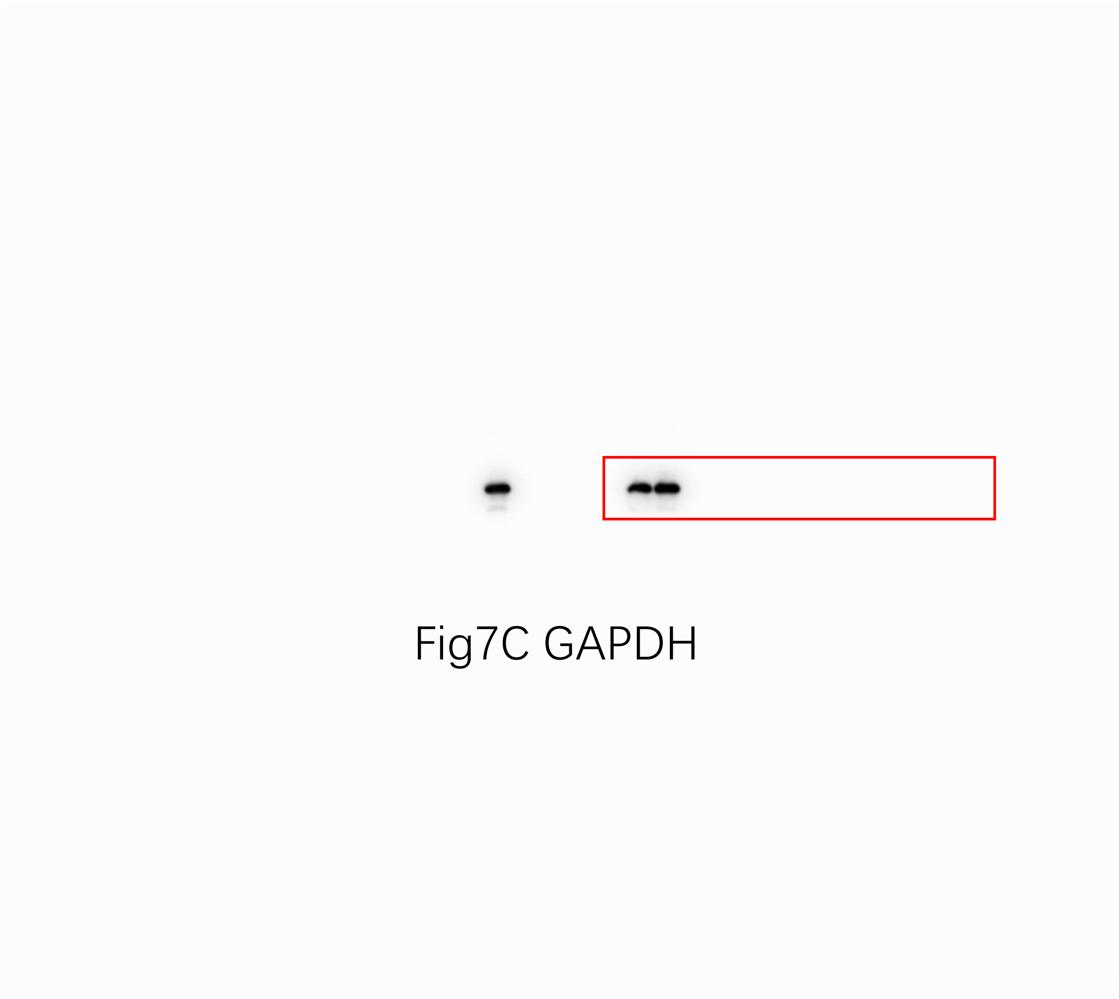

Fig7C GAPDH

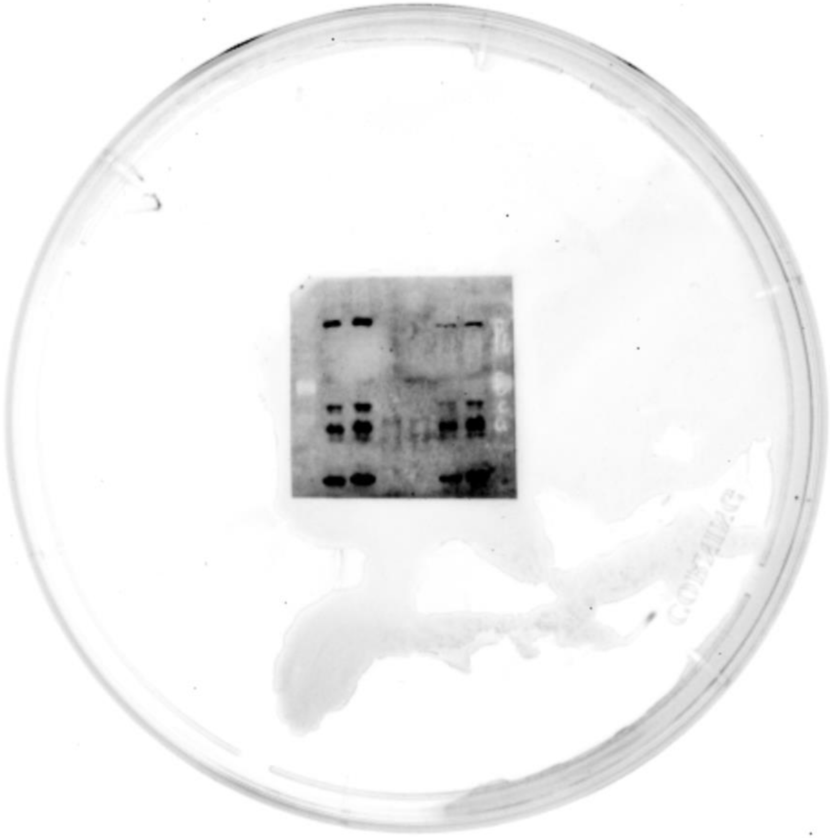

Fig7C Eca

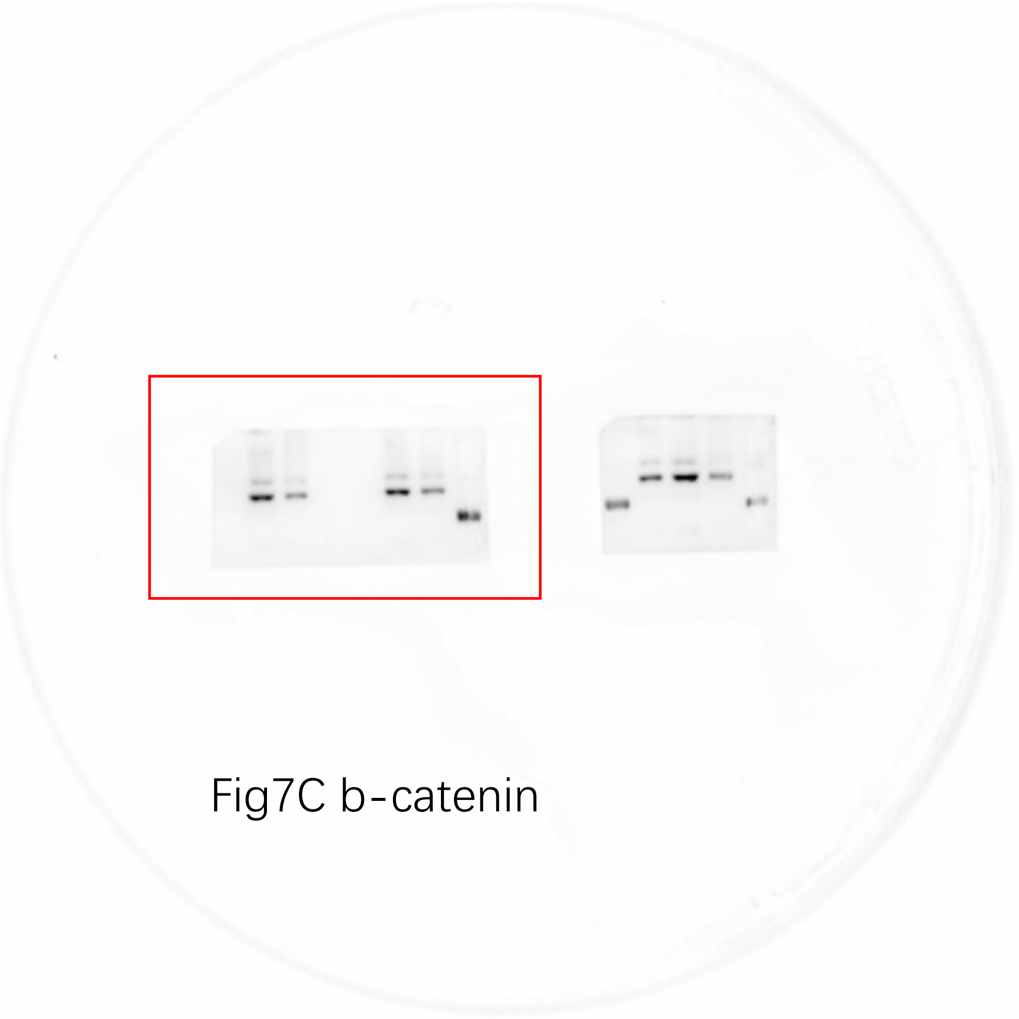

Fig7C b-catenin

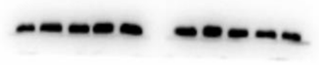

Fig7E GAPDH

Fig7E b-catenin

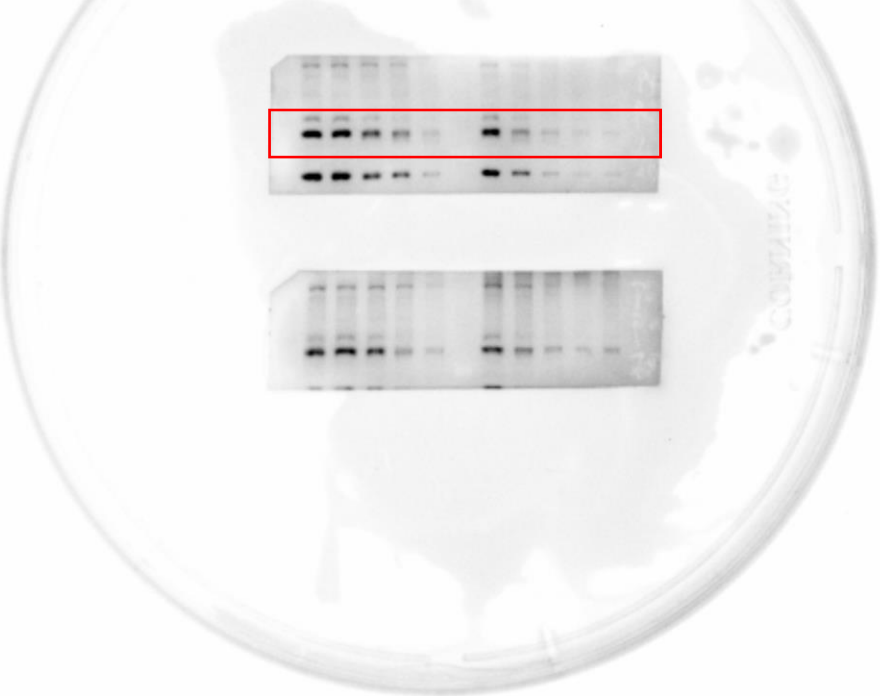

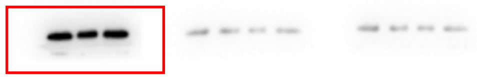

Fig7F GAPDH

Fig7G b-catenin

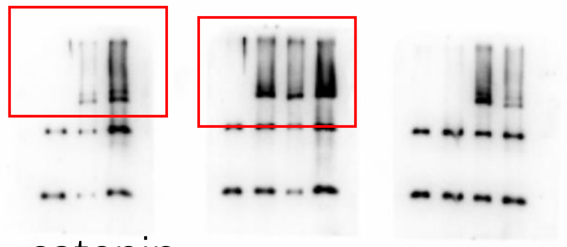

Fig7F b-catenin

Fig7G ub

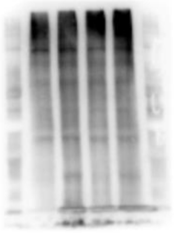

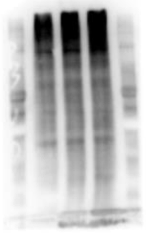

Fig7F ub

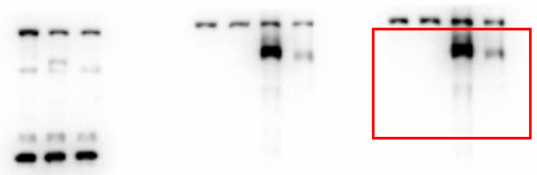

Fig7G Flag

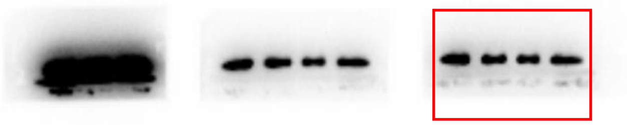

Fig7G GAPDH
